# SIRT2 Inhibition by AGK2 Promotes Perinuclear Cytoskeletal Organisation and Reduces Invasiveness of MDA-MB-231 Triple-Negative Breast Cancer Cells in Confined In Vitro Models

**DOI:** 10.3390/cells13232023

**Published:** 2024-12-07

**Authors:** Emily Jessop, Natalie Young, Beatriz Garcia-Del-Valle, Jack T. Crusher, Boguslaw Obara, Iakowos Karakesisoglou

**Affiliations:** 1 Department of Biosciences, Durham University, Durham DH1 3LE, UK; emilykjessop1@gmail.com (E.J.); natalie.young88@hotmail.com (N.Y.); beatrizgdv05@gmail.com (B.G.-D.-V.); jackcrusher@icloud.com (J.T.C.); 2 School of Computing, Newcastle University, Newcastle upon Tyne NE4 5TG, UK; boguslaw.obara@newcastle.ac.uk

**Keywords:** AGK2, lamin, LINC complex, microtubules, nesprins, nuclear mechanics, nucleus, SIRT2, vimentin

## Abstract

Triple-negative breast cancer (TNBC) is a highly aggressive breast cancer subtype characterised by the absence of targetable hormone receptors and increased metastatic rates. As nuclear softening strongly contributes to TNBC’s enhanced metastatic capacity, increasing the nuclear stiffness of TNBC cells may present a promising therapeutic avenue. Previous evidence has demonstrated the ability of Sirtuin 2 (SIRT2) inhibition to induce cytoskeletal reorganisation, a key factor in regulating nuclear mechanics. Thus, our study aimed to investigate the effect of SIRT2 inhibition on the nuclear mechanics and migratory behaviour of TNBC cells. To achieve this, SIRT2 was pharmacologically inhibited in MDA-MB-231 cells using AGK2, a SIRT2-specific inhibitor. Although SIRT2 inhibition had no effect on LINC complex composition, the AGK2-treated MDA-MB-231 cells displayed more prominent perinuclear organisations of acetylated α-tubulin, vimentin, and F-actin. Additionally, the nuclei of the AGK2-treated MDA-MB-231 cells exhibited greater resistance to collapse under osmotic shock. Scratch-wound assays also revealed that SIRT2 inhibition led to polarity defects in the MDA-MB-231 cells, while in vitro space-restrictive invasion assays highlighted their reduced migratory capacity upon AGK2 treatment. Taken together, our findings suggest that SIRT2 inhibition promotes a perinuclear cytoskeletal organisation in MDA-MB-231 cells, which enhances their nuclear rigidity and impedes their invasion through confined spaces in vitro.

## 1. Introduction

Breast cancer, the most commonly diagnosed female malignancy worldwide, is a highly heterogeneous disease that has been categorised into multiple subtypes [1,2]. Triple-negative breast cancer (TNBC) is a particularly aggressive breast cancer subtype that is responsible for 10–15% of all breast cancer diagnoses. Specifically, it is characterised by the lack of oestrogen receptor (ER) and progesterone receptor (PR) expression, as well as the loss of human epithelial growth receptor-2 (HER2) amplification [3,4,5]. Due to the lack of these specific molecular targets, effective treatment options against TNBC are limited and primarily depend on standard-of-care chemotherapy, to which 30% of patients develop resistance [6]. This results in poor overall survival and high rates of distant metastasis [7,8], with 45% of advanced-stage patients found to present brain or visceral metastases [9]. Consequently, novel therapeutic targets for TNBC patients are in great demand. 

Upon metastasis through the blood and lymphatic system, cancer cells must undergo major deformation to migrate through confined pores in the endothelium and stroma, which are often less than 2 μm in diameter [10,11,12]. However, with a diameter of 10–20 μm and greater stiffness compared to that of the cytoplasm, the nucleus presents a considerable challenge to cancer metastasis [13]. To overcome this physical barrier, metastatic cancer cells are known to increase their nuclear malleability [14], as highlighted by MDA-MB-231 and MCF-7 cells displaying softer nuclei than healthy breast epithelial cells [15]. Although the mechanisms by which cancer cells reduce their nuclear rigidity are incompletely understood, it is likely facilitated by breast tumour cells downregulating multiple LINC complex and nuclear lamina components involved in maintaining nuclear integrity [12,16]. Therefore, with nuclear softness strongly associated with heightened tumour aggressiveness [17,18], altering the nuclear mechanics of TNBC cells has the potential to impede their enhanced metastatic capacity [19].

The LINC (linker of the nucleoskeleton and cytoskeleton) complex is a highly conserved, nuclear envelope-spanning structure consisting of Nesprin and SUN domain proteins. It physically couples the nucleoskeleton to the cytoskeleton and is a major regulator of nuclear mechanics [20,21]. Humans express four Nesprin proteins (Nesprins 1-4), encoded by *SYNE1-4* [22]. These proteins exhibit a C-terminal KASH domain that anchors them to the outer nuclear membrane (ONM) by interacting with the SUN domain of SUN1 or SUN2 within the perinuclear space [23]. In addition, Nesprin proteins possess a central rod domain made up of variable numbers of spectrin repeats (SRs), as well as unique N-terminal domains, which bind specific cytoskeletal components [24,25]. The alternative splicing and transcription of *SYNE1* and *SYNE2* generate a plethora of Nesprin-1 and Nesprin-2 isoforms [21]. Full-length Nesprin-1 and Nesprin-2, referred to as the giant isoforms, are 1000 kDa and 800 kDa in molecular weight with 74 and 56 SRs, respectively [26]. Nesprin-1 giant and Nesprin-2 giant interact directly with filamentous actin (F-actin), through their highly conserved n-terminal actin-binding domain [27], and indirectly with the microtubule network via the microtubule motor proteins dynein and kinesin-1 [25,28,29]. Moreover, Nesprin-3α (encoded by *SYNE3*) possesses only eight SRs and binds the cytoskeletal linker protein, plectin, via its first SR, which recruits intermediate filaments to the ONM [30,31,32]. At the inner nuclear membrane (INM), however, SUN proteins interact with the nuclear lamina, a meshwork of type V intermediate filaments consisting of lamin A/C, lamin B1, and lamin B2, facilitating LINC–nucleoskeleton interactions and recruiting Nesprin proteins to the nucleus [23,33]. 

It is well established that perturbations in the expression of both LINC complex and nuclear lamina components play a key role in modulating nuclear stiffness. A depletion of Nesprin-1, Nesprin-2, SUN1, or SUN2 reduces nuclear rigidity [26,34,35], while an increase in lamin A/C expression induces nuclear stiffening [36,37,38,39]. Moreover, alterations in the cytoskeletal organisation are known to impact nuclear mechanics. Previous research has demonstrated that the loss of F-actin cables within the perinuclear actin cap leads to nuclear softening in mouse embryonic fibroblasts and, importantly, in TNBC cells [15,40]. Evidence also implicates keratin and vimentin intermediate filaments in maintaining nuclear stiffness [41,42,43]. Therefore, given that TNBC cells downregulate Nesprin-2, SUN1, SUN2, and lamin A/C expression [16], increasing the expression of LINC complex or nuclear lamina components, or reorganising the cytoskeleton around the nucleus, may increase the nuclear stiffness of TNBC cells.

Sirtuins (SIRTs) are a group of class III histone deacetylases (HDACs) that depend on nicotinamide dinucleotide (NAD^+^) for their activity and target both histone and non-histone proteins [44,45]. Mammals possess seven different sirtuin proteins (SIRT1-7), which display differing subcellular localisations and function in various cancer-associated processes, including DNA damage repair, cellular stress, and metabolism [46,47]. As a result, multiple SIRTs have been identified as promising targets for cancer therapy [48]. However, due to its prominent upregulation in TNBC, this study focused specifically on the NAD^+^-dependent deacetylase Sirtuin 2 (SIRT2) [49,50,51]. Although SIRT2 deacetylates nuclear targets, such as histone 4 (H4), forkhead box O3a (FOXO3a), and homeobox protein Hox-A10 (HOXA10) [52,53,54], regulating chromatin condensation and transcription, it is primarily localised in the cytoplasm [55]. Here, SIRT2 deacetylates lysine 40 of α-tubulin [56,57] and lysine 207 of keratin 8 [58]. Consequently, SIRT2 regulates the acetylation and organisation of these cytoskeletal networks. More specifically, pharmacological inhibition of SIRT2 using AGK2, a competitive inhibitor that binds to the C-site of SIRT2, blocking NAD^+^ binding and inhibiting the deacetylase activity of SIRT2 [59,60,61], has been shown to predominantly affect the perinuclear α-tubulin and keratin 8 networks. This is supported by previous evidence showing that AGK2 treatment results in the strong hyperacetylation of perinuclear α-tubulin in HeLaS3 cells and induces a more pronounced perinuclear keratin 8 organisation, along with the formation of aggregates, in HepG2 hepatocarcinoma cells [58,62]. With the perinuclear cytoskeletal organisation contributing significantly to nuclear mechanics [15,40,42], this evidence suggests that SIRT2 inhibition has the potential to modulate nuclear rigidity. This is strongly supported by the ability of SIRT2-knockdown or SIRT2 inhibition, using salermide, to impede the metastasis of hepatocellular carcinoma, gastric cancer, and osteosarcoma cells in xenograft mouse models [63,64,65]. However, these studies did not explore the potential for SIRT2 inhibition to modify the cytoskeletal organisation or LINC complex composition of these cancer types. 

Thus, the current work aimed to investigate the potential for SIRT2 inhibition to modulate the nuclear mechanics and migratory capacity of TNBC cells, focusing on the underlying mechanisms promoting the observed changes. This was achieved by treating the highly invasive TNBC line MDA-MB-231 with the potent SIRT2-specific inhibitor AGK2, which has minimal effects on other sirtuins at concentrations <40 μM [59]. In particular, this study explored the effect of SIRT2 inhibition on the organisation of the microtubule, intermediate filament, and actin cytoskeletal networks, as well as the expression of core LINC complex and nucleoskeletal components. In addition, the impact of AGK2 treatment on the nuclear rigidity and migratory capacity of MDA-MB-231 cells was explored through osmotic shock and in vitro scratch-wound and space-restrictive migration assays. Overall, this work demonstrates the ability of AGK2-induced SIRT2 inhibition to increase the nuclear stiffness and dampen the invasiveness of MDA-MB-231 cells, whilst providing novel insights into the associated mechanisms. 

## 2. Materials and Methods

### 2.1. MDA-MB-231, MCF10A, and HCC38 Cell Culture

The MDA-MB-231 cell line (item number 92020424) was obtained from the European Collection of Authenticated Cell Cultures (ECACC, Salisbury, UK), whilst MCF10A cells (CRL-10317) and HCC38 cells (CRL-2314) were procured from the American Type Culture Collection (ATCC, Manassas, VA, USA). All cell lines were routinely cultured as cell monolayers in phenol red-free media, and incubated under humidified conditions at 37 °C and 5% CO_2_ (PHCbi cell culture incubator, Loughborough, UK). The individual conditions for each cell line are indicated below. 

MDA-MB-231 cells were cultured in DMEM medium (4.5 g/L glucose; Corning, Flintshire, UK), supplemented with 10% foetal bovine serum (FBS; Sigma-Aldrich, Gillingham, UK), 1% penicillin–streptomycin antibiotics (Sigma-Aldrich, Gillingham, UK), and 2 mM L-Glutamine (Sigma-Aldrich, Gillingham, UK). 

MCF10A cells were cultured in DMEM/F-12 medium (Sigma-Aldrich, Gillingham, UK), supplemented with 5% (*v/v*) horse serum (Invitrogen, Loughborough, UK), 1% penicillin–streptomycin antibiotics (Sigma-Aldrich, Gillingham, UK), 100 ng/mL cholera toxin (Sigma-Aldrich, Gillingham, UK), 20 ng/mL epidermal growth factor (Peprotech, ThermoFisher Scientific, Cramlington, UK), 2 mM L-Glutamine (Sigma-Aldrich, Gillingham, UK), 500 ng/mL hydrocortisone (Sigma-Aldrich, Gillingham, UK), and 0.01 mg/mL insulin (Sigma-Aldrich, Gillingham, UK).

HCC38 cells were cultured in RPMI medium 1640 (Gibco, ThermoFisher Scientific, Cramlington, UK) supplemented with 10% foetal bovine serum (FBS; Sigma-Aldrich, Gillingham, UK), 1% penicillin–streptomycin antibiotics (Sigma-Aldrich, Gillingham, UK), and 2 mM L-Glutamine (Sigma-Aldrich, Gillingham, UK).

Cells underwent routine enzymatic passaging using 0.05% Trypsin-EDTA (Sigma-Aldrich, Gillingham, UK). After removing the cell growth media, the cells were rinsed twice with Dulbecco's Phosphate-Buffer Saline (DPBS; Gibco, ThermoFisher Scientific, Cramlington, UK). Trypsin-EDTA solution at 0.05% was added to the cell monolayer and incubated (MDA-MB-231, 10 min; MCF10A, 20 min; HCC38, 15 min) under humidified conditions at 37 °C with 5% CO_2_. Complete cell detachment was confirmed using a Zeiss Telaval 31 light microscope (Carl Zeiss Ltd, Cambridge, UK). Fresh medium was added to the suspended cells, and the resulting mixture was transferred into a Falcon tube. Following this, the cell suspension was centrifuged at 1000× *g* for 5 min (Fisherbrand^TM^ Centrifuge GT2, ThermoFisher Scientific, Cramlington, UK) to pellet the cells and the supernatant was carefully discarded before re-suspending the cell pellet in fresh medium. The Countess 3^TM^ automated cell counting device (ThermoFisher Scientific, Cramlington, UK) was implemented to calculate cell numbers. 

Cells were treated with AGK2 (Cayman Chemical, Ann Arbor, MI, USA) for the pharmacological inhibition of Sirtuin 2 (SIRT2). A 20 mM AGK2 stock solution was prepared in DMSO (Dimethyl Sulfoxide; ThermoFisher Scientific, Cramlington, UK) for subsequent dilution to a final concentration of 5 μM AGK2 prior to treatment. The cells were subjected to 5 μM AGK2 treatment for 24 h at 37 °C and 5% CO_2_ in a cell incubator with controlled humidity.

### 2.2. SDS-PAGE and Western Blotting

Cell culture flasks were washed twice with ice-cold PBS (Severn Biotech, Kidderminster, UK) before adding ice-cold RIPA lysis buffer (50 mM Tris, 150 mM NaCl, 0.1% SDS, 1% Nonidet P-40, 0.5% Sodium-deoxycholate, 1% Protein Inhibitor Cocktail P2714 (Sigma-Aldrich, Gillingham, UK) and incubating on ice for 15 min. Subsequently, the cells were harvested with a cell scraper (Corning, Flintshire, UK), sonicated at 40 kHz for 5 min (Branson 1510 Ultrasonic Cleaner), and centrifuged at 17,000× *g* (Heraeus Fresco 17 Centrifuge, ThermoFisher Scientific, Loughborough, UK) at 4 °C for 10 min. After determining the protein concentration of the supernatant using the BCA Protein Assay kit (ThermoFisher Scientific, Cramlington, UK), the sample was mixed with sample loading buffer (5 x Laemmli buffer, containing 5% β-mercaptoethanol) at a 1:5 ratio and denatured at 99 °C for 4 min. 

For protein separation, the samples were loaded onto 8% SDS-PAGE gels or, when targeting proteins larger than 250 kDa, Novex^TM^ WedgeWell^TM^ 4–12% Tris-Glycine gradient gels (Invitrogen, Loughborough, UK) were implemented. After SDS-PAGE, equal protein loading was confirmed by staining the gels with Coomassie InstantBlue^®^ (Abcam, Cambridge, UK) for 1 h at room temperature. To analyse protein expression, proteins were transferred from the polyacrylamide gel to a PVDF membrane (Immobilon-P, Merck Millipore, Burlington, MA, USA) activated with methanol, by utilising a semi-dry Invitrogen Power Blotter device (ThermoFisher Scientific, Loughborough, UK). This was achieved by placing the PVDF membrane and pre-run SDS-PAGE gel between two layers of filter paper (Whatman 3MM), creating the typical transfer stack. Prior to transfer, the PVDF membrane and the filter papers of the transfer stack were submerged in Power Blotter 1-Step Transfer Buffer (1x; ThermoFisher Scientific, Loughborough, UK). Finally, the ‘high-molecular-weight proteins’ pre-set method (1.3A for 10 min) was selected to complete protein transfer. Subsequently, the PVDF membrane was placed in methanol for 10 s and allowed to air-dry (minimum 15 min) before being blocked in 5% *w/v* non-fat dry milk solution in PBS for 1 h at room temperature. The membrane was then rinsed twice with PBS and washed for 5 min with PBS-Tween (pH 7.4, 0.1% Tween 20). 

For immunoblotting, the PVDF membranes were probed with primary antibody (as listed in Table S1) diluted in 5% milk (PBS) overnight at 4 °C, followed by three 10 min washing steps using PBS-Tween (pH 7.4, 0.1% Tween 20). Subsequently, the membrane was incubated with the appropriate horseradish peroxidase-conjugated (HRP) secondary antibody (as listed in Table S2) for 1 h at room temperature. The membrane was then washed three times for 5 min each in PBS-Tween (pH 7.4, 0.3% Tween 20), followed by three 5 min washes in PBS-Tween (pH 7.4, 0.1% Tween 20). Finally, Clarity^TM^ Western ECL substrate (BioRad, Watford, UK) was mixed in a 1:1 ratio and added to the membrane, which was then incubated in the dark for 5 min. The ECL signals were subsequently detected using the iBright 1500 imaging platform (ThermoFisher Scientific, Loughborough, UK) on the ChemiBlot setting, ensuring the exposure time did not induce band saturation. Protein expression levels were calculated by densitometric analysis, using Fiji, and normalised to the GAPDH loading control. 

### 2.3. Immunofluorescence Staining and Microscopy Imaging

Cells were seeded onto glass coverslips (11 mm, LaboQuip, London, UK) and cultured until they reached a confluency of 60–70%. After rinsing the coverslips twice with 1 x BRB80 buffer (80 mM K-PIPES (ThermoFisher Scientific, Cramlington, UK), 1 mM EGTA (Sigma-Aldrich, Gillingham, UK), 1 mM MgCl_2_ (Sigma-Aldrich, Gillingham, UK), adjusted to pH 6.8 with KOH (ThermoFisher Scientific, Cramlington, UK)), the cells underwent fixation and permeabilisation using 3.7% formaldehyde and 0.5% Triton X-100 in 1x BRB80 solution for 20 min in an incubator at 37 °C. Subsequently, the cells were washed with PBS buffer (Severn Biotech, Kidderminster, UK) and treated with phosphate-buffered gelatine solution (PBG; 0.1% fish gelatine (Sigma-Aldrich, Gillingham, UK), 1% bovine serum albumin (BSA; Sigma-Aldrich, Gillingham, UK), and 0.1% Triton X-100 (ThermoFisher Scientific, Cramlington, UK)) for 1 h at room temperature.

The coverslips were then incubated in primary antibody (as listed in Table S3) diluted in PBG for 1 h at ambient temperature, followed by three 5 min washes and one 15 min wash in PBS solution. Afterwards, the cells were treated with the relevant secondary antibody (as listed in Table S4) diluted in PBG for 1 h at ambient temperature in the dark. The PBS washes described previously were then repeated before counterstaining cells with DAPI solution (2 μg/mL in PBS; Sigma-Aldrich, Gillingham, UK) for 5 min at ambient temperature, shielded from light. Finally, the cells were rinsed twice using PBS solution (each wash lasting 5 min), before mounting the coverslips onto glass slides (LaboQuip, London, UK) using VECTASHIELD^®^ Antifade Mounting Medium (2bscientific, Bicester, UK). Immunofluorescence staining was captured using the Axioskop 40 epifluorescent microscope (Carl Zeiss Ltd, Cambridge, UK) or the Zeiss 880 Laser Scanning Confocal Microscope (LCSM; Carl Zeiss Ltd, Cambridge, UK). 

### 2.4. MTT Cytotoxicity Assay

MDA-MB-231 cells were seeded on a 96-well plate (Starlab, Milton Keynes, UK), at a density of 4000 cells per well, and incubated for 24 h in a humidified cell incubator at 37 °C and 5% CO_2_ to allow cell adhesion. Afterwards, the media were exchanged with fresh complete media (containing FBS) or serum-free media (lacking FBS). Next, the 96-well cell culture plate was incubated for 24 h at 37 °C and 5% CO_2_. The cells were subsequently treated with either 1 μM, 5 μM, or 10 μM AGK2 and incubated for another 24 h at 37 °C and 5% CO_2_. MTT (3-(4,5-Dimethylthiazol-2-yl)-2,5-Diphenyltetrazolium Bromide) (ThermoFisher Scientific, Cramlington, UK) was mixed with the existing media at a 1:1 ratio, resulting in a final MTT concentration of 0.5 mg/mL. The cells were treated with MTT for 3 h at 37 °C and 5% CO_2_. After discarding the MTT solution from each well, the resulting formazan crystals were solubilised in DMSO (ThermoFisher Scientific, Cramlington, UK). The absorbance of each well was recorded at 570 nm using a microplate reader (BioTek ELx800, Swindon, UK). All experiments were performed in triplicate. 

### 2.5. Osmotic Shock Assay

MDA-MB-231 cells were seeded onto glass coverslips (11 mm, LaboQuip, London, UK) and cultured until they reached a confluency of 60%. The cell culture medium was then replaced with fresh medium containing sucrose (640 mOsm), and the cells were placed in a humidified cell incubator at 37 °C and 5% CO_2_ for 30 min. Next, the cells were fixed, permeabilised, and blocked, before being processed for immunofluorescence labelling using lamin A/C as a nuclear envelope marker, following the protocol detailed in Section 2.3. The coverslips were then imaged using the Axioskop 40 epifluorescent microscope (Carl Zeiss Ltd, Cambridge, UK) to visualise nuclear envelope folds. 

### 2.6. EdU Cell Proliferation Assay 

The EdU cell proliferation assay was performed using the Click-iT^®^ EdU imaging kit according to the manufacturer’s instructions (ThermoFisher Scientific, Cramlington, UK). In summary, 20 μM EdU was mixed at a 1:1 ratio with fresh media and added to MDA-MB-231 cells, achieving a final 10 μM concentration of EdU. The cells were then placed in a humidified cell culture incubator at 37 °C and 5% CO_2_ for incubation. After 45 min, the medium was discarded, and the cells were rinsed with PBS buffer (Severn Biotech, Kidderminster, UK) before being treated with 4% paraformaldehyde PBS buffer for 20 min (fixation) at ambient temperature. Following this, the cells were permeabilised with 0.5% Triton X-100 in PBS for 20 min at room temperature and subsequently rinsed using 3% BSA/PBS solution. Subsequently, 500 μL of the Click-iT^®^ reaction mixture was added to the cells and incubated for 30 min at room temperature in the dark. After rinsing the cells with 3% BSA/PBS, the cells were then incubated with Hoechst solution for 30 min at room temperature, protected from light. Finally, VECTASHIELD^®^ Antifade Mounting Medium (2bscientific, Bicester, UK) was used to mount the coverslips onto glass slides (LaboQuip, London, UK). 

### 2.7. Two-Dimensional Scratch-Wound Assay

Cells were plated on 12-well culture plates (Starlab, Milton Keynes, UK) and cultured until they reached 100% confluency. The cells were incubated in serum-free media for 24 h prior to the experiment at 37 °C and 5% CO_2_ in a humidified cell incubator (PHCbi, Loughborough, UK). Immediately before starting the migration assay, a vertical scratch in the monolayer was created at the centre of each well using a 200 μL pipette tip (Starlab, Milton Keynes, UK), and the cells were exposed to 5 μM AGK2. Live cell migration was observed using the Zeiss Cell Observer microscope (Carl Zeiss Ltd, Cambridge, UK) in an environmental chamber set at 37 °C and 5% CO_2_. Images were taken at 15 min intervals for 24 h.

### 2.8. Cell Polarisation Assay

MDA-MB-231 cells were seeded onto glass coverslips (13 mm, LaboQuip, London, UK) and cultured until they reached 100% confluence. Using a 200 μL pipette tip (Starlab, Milton Keynes, UK), a vertical scratch was made at the centre of each monolayer and the cells were incubated for 2 h at 37 °C and 5% CO_2_, enabling cell polarisation to occur. The cells were fixed, permeabilised, and blocked following the protocol described in Section 2.3, before being incubated with GM130 primary antibody (BD Biosciences, Wokingham, UK) in order to document the migration-induced polarisation of the Golgi apparatus. Subsequently, the cells were labelled with TRITC-phalloidin (20 ng/mL; Sigma-Aldrich, Gillingham, UK) and the corresponding secondary antibody. For microscopy imaging, the coverslips were examined using the Axioskop 40 epifluorescent microscope (Carl Zeiss Ltd, Cambridge, UK). 

### 2.9. Space-Restrictive Cell Migration Chemotaxis Assays

The cells were serum-starved for 24 h before starting the experiment. Following this, the cells were harvested and counted as outlined in Section 2.1. Plates and reagents from the invasion assay (ECM555, 8 μm porous membranes, Sigma-Aldrich, Gillingham, UK) were equilibrated at ambient temperature for 2 h before the start of the assay. Plates were disassembled and 100 μL of prewarmed (37 °C) serum-free media was supplied to each well containing the ECMatrix^TM^ membrane (cell invasion chamber plate) to rehydrate the ECM layer. The setup was left for 1 h at room temperature, after which the media were carefully removed. A volume of 150 μL of prewarmed complete media was supplied to each well of the feeder tray, and the plate was reassembled. A total of 20,000 cells in 100 μL of serum-free media, along with pharmacological treatment, were added to each well of the cell invasion chamber plate. The lid was placed on the plate, and the cells were incubated for 24 h in a humidified chamber at 37 °C and 5% CO_2_. After incubation, the media and cells were removed from the top chamber by pipetting. The top chamber was then rinsed by placing it on top of a new feeder tray with 150 μL of Dulbecco's Phosphate-Buffer Saline (DPBS; Gibco, ThermoFisher Scientific, Cramlington, UK) in each well for 1 min at room temperature. The PBS was discarded and, to aid the dislodgment of cells, 150 μL of prewarmed cell detachment solution was added to the wells of the feeder tray. The plate was assembled and allowed to incubate for 30 min at 37 °C. During the incubation period, the plate was manually rocked to help dislodge cells. After 30 min, the plate was disassembled, the invasion chamber plate was discarded, and 50 μL of lysis buffer/dye solution (CyQuant GR Dye was added at a 1:75 ratio to 4X lysis buffer) was added to the 150 μL cell detachment solution in each well of the feeder tray and the plate was incubated for a further 15 min at room temperature. A volume of 150 μL of solution was then transferred from each well of the feeder tray to the wells of a 96-well plate (Starlab, Milton Keynes, UK). Fluorescence measurements were taken at 520 nm using a GloMax Discover microplate reader (Promega, Chilworth, UK) equipped with a 480/520 nm filter set.

The cell migration assay (96-well format) using 5 μm porous membranes (ab235693, Abcam, Cambridge, UK) was performed following the manufacturer’s instructions.

### 2.10. Statistical Analysis

The data were analysed using Microsoft Excel version 16.63.1, IBM^®^ SPSS^®^ Statistics version 28.0.0.0, and GraphPad Prism version 9 (GraphPad, San Diego, CA, USA). The statistical significance between two groups was determined using a Student’s unpaired *t*-test. To analyse more than two groups, a one-way ANOVA (Analysis of Variance), followed by a post hoc Dunnett’s test, was implemented to determine statistical significance compared to the ‘vehicle’ control. The error bars represent ± SEM (standard error of the mean), and significance was determined at a *p*-value of ≤0.05. The level of significance between groups is demonstrated by ns, no significance, * *p* ≤ 0.05, ** *p* ≤ 0.01, *** *p* ≤ 0.001, and **** *p* ≤ 0.0001.

## 3. Results

### 3.1. AGK2 Treatment Leads to More Prominent Perinuclear Networks of Acetylated α-Tubulin and Vimentin in MDA-MB-231 Cells

To determine a suitable concentration of AGK2 for treating MDA-MB-231 cells, an MTT assay was first conducted to assess potential cell toxicity effects. This enabled us to determine the viability of MDA-MB-231 cells following a 24 h treatment with a range of AGK2 concentrations (1 μM, 5 μM, and 10 μM) (Figure S1). However, as the MTT test also measures cell proliferation and metabolic activity, the assay was performed in both complete media (with FBS) and serum-free media (without FBS) to examine the impact of AGK2 in non-proliferating cells. In complete media, all three AGK2 concentrations significantly decreased the viability of the MDA-MB-231 cells (Figure S1A). While the treatment with 10 μM AGK2 reduced the cell viability to 47.4%, the treatment with 1 μM and 5 μM AGK2 only lowered the cell viability to 70.7% and 64.3%, respectively. In contrast, the cell viability remained above 80% when the MDA-MB-231 cells were treated with 1 μM, 5 μM, or 10 μM AGK2 in serum-free media (Figure S1B), indicating minimal toxicity to MDA-MB-231 cells. Therefore, we ultimately selected 5 μM AGK2 for a 24 h treatment of the MDA-MB-231 cells. 

**Figure 1:**
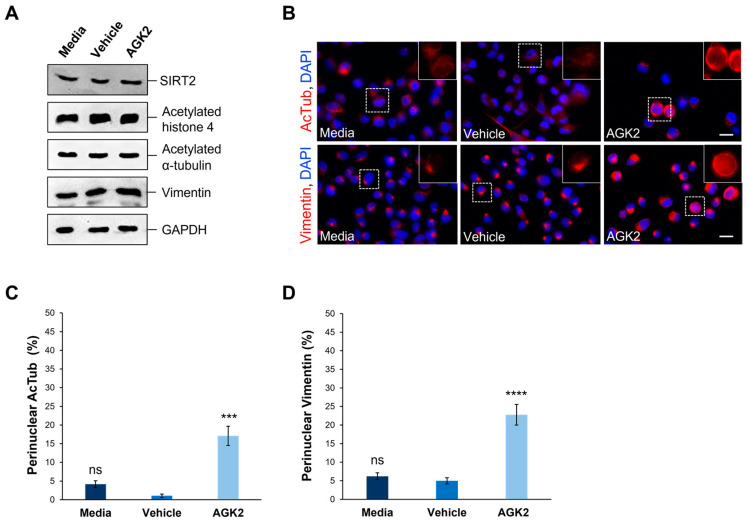
AGK2-induced SIRT2 inhibition promotes the perinuclear localisation of acetylated α-tubulin and vimentin in MDA-MB-231 cells. (**A**) Western blot analysis showing SIRT2, acetylated histone 4, acetylated α-tubulin, and vimentin protein expression levels in media-, vehicle-, and AGK2-treated MDA-MB-231 cells. GAPDH expression is used to demonstrate equal protein loading. (**B**) Immunofluorescence microscopy images of acetylated α-tubulin (AcTub, upper row) and vimentin (lower row) organisation in control (media and vehicle) and AGK2-treated MDA-MB-231 cells. Nuclei are visualised using a DAPI counterstain (blue channel). Insets (showing only the red channel) are higher magnifications of the areas indicated by dashed boxes. Scale bar: 20 μm. (**C**,**D**) Quantification of the percentage (%) of control and AGK2-treated cells exhibiting prominent perinuclear acetylated α-tubulin (**C**) and vimentin (**D**) rings; >350 cells analysed for each condition. The data are presented as the mean ± SEM (standard error of the mean). Statistical significance was determined using a one-way ANOVA followed by a Dunnett’s post hoc test to compare with the vehicle control. “ns” denotes no significant difference, while *** *p* ≤ 0.001 and **** *p* ≤ 0.0001 indicate statistical significance.

Subsequently, given that AGK2 is a SIRT2-specific inhibitor, Western blotting was implemented to investigate the effect of a 24 h treatment with 5 μM AGK2 on the SIRT2 protein levels in the MDA-MB-231 cells (Figure 1A). Prior to this, equal protein loading of cell lysates was confirmed by carrying out an InstantBlue Coomassie stain on the gel following SDS-PAGE (Figure S2A). Moreover, the expression levels of ubiquitous proteins (β-tubulin, β-actin, and GAPDH) did not vary significantly across the conditions (Figure S2B–E). The densitometric quantification of the SIRT2 protein levels revealed no significant difference between the AGK2-treated and control (media-treated and vehicle-treated) MDA-MB-231 cells (Figure S3A). Consequently, we investigated the expression of SIRT2 downstream targets, histone 4 and α-tubulin, by immunoblotting against acetylated histone 4 and acetylated α-tubulin (Figure 1A). Similarly to SIRT2, the AGK2 treatment did not significantly alter the levels of these proteins in the MDA-MB-231 cells compared to controls (Figure S3B,C). 

Given the previous evidence demonstrating the ability of SIRT2 inhibition to impact the organisation of acetylated α-tubulin and keratin 8, immunofluorescence staining was employed to assess the effect of AGK2 on the organisation of these cytoskeletal networks. In media-treated and vehicle-treated MDA-MB-231 cells, acetylated α-tubulin displayed a weak perinuclear organisation, which often dispersed into the cytoplasm. In contrast, the AGK2-treated cells exhibited more prominent acetylated α-tubulin staining around the nucleus (Figure 1B, upper row). When examining the keratin 8/18 organisation, it was observed that the MDA-MB-231 cells lacked an extensive network of this intermediate filament component, with the control and AGK2-treated cells displaying only a singular aggregation at the nuclear periphery (Figure S4A). This subsequently prompted an investigation of the keratin 8/18 expression (Figure S4B), with the Western blot revealing no significant changes in the expression of keratin 8 or keratin 18 between the control and AGK2-treated MDA-MB-231 cells (Figure S4C,D). Therefore, as an alternative intermediate filament marker, the expression and organisation of vimentin in MDA-MB-231 cells was examined next. Whilst SIRT2 inhibition did not affect the vimentin expression (Figure 1A and Figure S3D), the AGK2-treated cells displayed more prominent perinuclear vimentin rings compared to the control cells, which exhibited a tight aggregation adjacent to the nucleus (Figure 1B, lower row). This led to the quantification of cells displaying perinuclear acetylated α-tubulin and perinuclear vimentin networks, with the insets in Figure 1B highlighting the perinuclear phenotype presented by AGK2-treated MDA-MB-231 cells. Whilst 4.16% of the media-treated and 1.05% of the vehicle-treated cells exhibited a perinuclear acetylated α-tubulin ring, this was significantly increased to 17% when the cells received a 24 h treatment with 5 μM AGK2 (Figure 1C). Similarly, the AGK2 treatment induced a significant increase in the percentage of perinuclear vimentin rings from 6.2% in the media-treated and 5% in the vehicle-treated cells, to 22.7% in the AGK2-treated cells (Figure 1D). Collectively, the evidence suggests that AGK2 treatment elicits significant changes in the organisation of acetylated-tubulin and vimentin around the nucleus in MDA-MB-231 cells. 

### 3.2. AGK2-Induced SIRT2 Inhibition Promotes Significant Perinuclear F-actin Ring Formation in a Small Subset of MDA-MB-231 Cells

**Figure 2:**
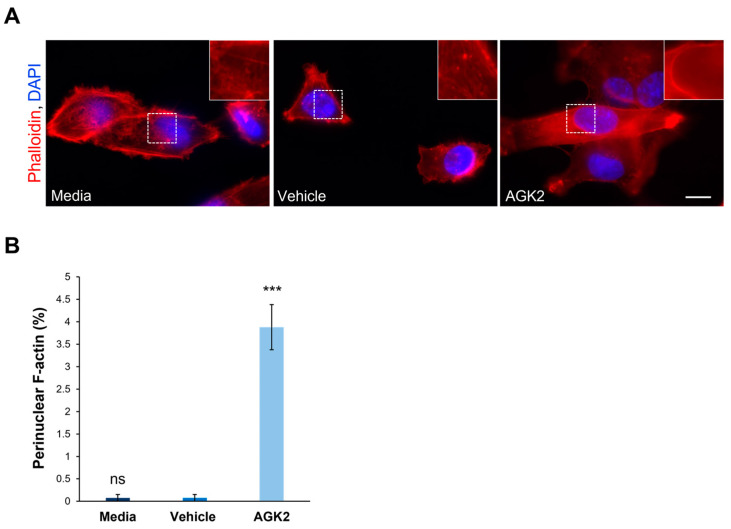
Pharmacological inhibition of SIRT2 in MDA-MB-231 cells induces the formation of perinuclear F-actin rings. (**A**) Representative fluorescence microscopy images of filamentous actin (F-actin) organisation in control and AGK2-treated MDA-MB-231 cells stained with TRITC-phalloidin (red channel) and DAPI (blue channel). Insets (showing only the red channel) are higher magnifications of the areas marked by dashed boxes. Scale bar: 10 μm. (**B**) Quantification of the percentage (%) of cells that displayed a perinuclear F-actin ring; >800 cells analysed per condition. The data are presented as the mean ± SEM, and statistical analysis was determined using a one-way ANOVA and Dunnett’s post hoc test for comparisons with the vehicle control. “ns” indicates no significance while *** *p* ≤ 0.001 denotes statistical significance.

Following the finding that AGK2 treatment impacts the microtubule and vimentin networks, the effect of SIRT2 inhibition on the organisation of filamentous actin (F-actin) in MDA-MB-231 cells was explored. This was achieved using fluorescently labelled phalloidin, which specifically binds F-actin and allows for the visualisation of F-actin-based structures. The F-actin network was spread throughout the cytoplasm of the control (media- and vehicle-treated) and AGK2-treated MDA-MB-231 cells, with strong staining localised at the cell cortex (Figure 2A). However, the AGK2 treatment also led to the formation of prominent perinuclear F-actin rings (see inset in Figure 2A) in a small subset of MDA-MB-231 cells, as highlighted by the lower-magnification images in Figure S5A. Despite quantification revealing that only 3.88% of the AGK2-treated cells exhibited a perinuclear F-actin ring, this was a significant increase compared to that in the control cells, in which these F-actin structures were largely absent (Figure 2B). Therefore, these results demonstrate that AGK2-induced SIRT2 inhibition affects all the major constituents of the cytoskeleton, including the microtubule, intermediate filament, and actin networks. Given the cytoskeleton’s role in determining nuclear shape, a morphometric analysis of the nuclear area and contour ratio (a measure of nucleus circularity) was executed in the media-, vehicle- and AGK2-treated cells. As demonstrated in Figure S6, however, the AGK2 treatment did not affect the shape or size of the nucleus. 

### 3.3. SIRT2 Inhibition Does Not Significantly Impact the Expression or Organisation of Nesprin-1 Giant or Nesprin-2 Giant Molecules

**Figure 3:**
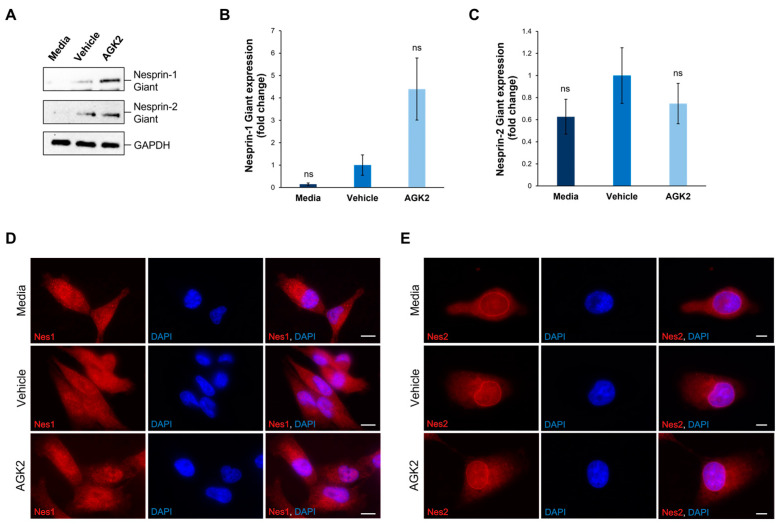
The expression and localisation of Nesprin-1 giant and Nesprin-2 giant are unaffected in AGK2-treated MDA-MB-231 cells. (**A**) Immunoblot analysis of Nesprin-1 giant and Nesprin-2 giant protein expression in media-, vehicle-, and AGK2-treated MDA-MB-231 cells. GAPDH expression demonstrates equal protein loading. (**B**,**C**) Densitometric quantification of Nesprin-1 giant (**B**) and Nesprin-2 giant (**C**) protein expression, normalised against the GAPDH loading control and presented as an average fold change relative to the vehicle treatment. The data are presented as the mean ± SEM, n = 3. Statistical analysis was conducted using a one-way ANOVA, and no significant differences (indicated by “ns”) were observed between the media- and AGK2-treated groups, compared to the vehicle-treated control. (**D**) Immunofluorescence microscopy images of control and AGK2-treated MDA-MB-231 cells stained with N1ABD antibody (red channel), specific to the actin-binding domain of Nesprin-1 (Nes1). Nuclei are visualised using DAPI counterstain (blue channel). Scale bar: 10 μm. (**E**) Microscopy images of control and AGK2-treated MDA-MB-231 cells immunostained with pAbK1 antibody (red channel), specific to the C-terminus of Nesprin-2 (Nes2). DAPI indicates nuclear staining. Scale bar: 5 μm.

To gain insights into the underlying mechanisms causing the changes in the peri-nuclear cytoskeletal organisation upon AGK2 treatment, the expression and organisation of key LINC complex components, Nesprin-1 and Nesprin-2, were examined using Western blotting and immunofluorescence staining (Figure 3). The focus was specifically on the giant isoforms of these Nesprin proteins (>800 kDa) due to their ability to interact directly with the actin cytoskeleton, through their N-terminal actin-binding domain (ABD), and indirectly with microtubule filaments, via crosslinks with microtubule motor proteins. For the analysis of Nesprin-1, rabbit polyclonal antibodies (N1ABD) targeting the Nesprin-1 actin-binding domain (ABD) were used. For Nesprin-2, rabbit polyclonal antibodies (pAbK1) detecting the C-terminus of Nesprin-2 giant, which encompasses spectrin repeats 55 and 56, were employed. Although the expression of Nesprin-1 giant increased 4.4-fold upon AGK2 treatment relative to the vehicle control, the statistical analysis confirmed that this change was not significant (Figure 3A,B). However, the p-value was calculated as 0.051, indicating that the result could be considered marginally significant, particularly given the technical challenges with consistently detecting Nesprin-1 giant and Nesprin-2 giant. This could also explain the large error bars when quantifying the expression of Nesprin-2 giant, in which there was no significant difference between the control and AGK2-treated cells (Figure 3A,C).

Subsequently, the impact of AGK2 treatment on the localisation of Nesprin-1 and Nesprin-2 was assessed, as alterations in the distribution of these proteins could affect the organisation of the associated cytoskeletal networks. In contrast to the Western blotting experiments, the immunofluorescence staining was not focused specifically on Nesprin-1 giant and Nesprin-2 giant, as the antibodies used in these experiments detected multiple Nesprin-1 and Nesprin-2 isoforms. Thus, Figure 3D,E represent the distribution of the smaller Nesprin-1 and Nesprin-2 proteins, as well as their giant isoforms. Surprisingly, in both the control and AGK2-treated MDA-MB-231 cells, Nesprin-1 was not localised to the nuclear envelope, but rather exhibited a dotty pattern throughout the cytoplasm (Figure 3D). On the other hand, Nesprin-2 was predominantly localised to the nuclear envelope in the media-, vehicle-, and AGK2-treated cells, with some staining observed within the cytoplasm (Figure 3E). Together, these findings demonstrate that AGK2 has no major effects on the protein levels or organisation of Nesprin-1 and Nesprin-2. This suggests that an alternative mechanism is responsible for the perinuclear cytoskeletal enrichment exhibited in the AGK2-treated MDA-MB-231 cells, particularly as Nesprin-1 was not detected at the nuclear envelope. 

Intrigued by the lack of Nesprin-1 (N1ABD) nuclear envelope staining in the media-, vehicle-, and AGK2-treated MDA-MB-231 cells, further immunofluorescence staining for Nesprin-1 was performed using an additional anti-Nesprin-1 antibody. Specifically, a rabbit polyclonal antibody, termed specII, which targets the C-terminus of Nesprin-1 giant, encompassing spectrin repeats 73 and 74, was employed. Similarly to the N1ABD antibody, the specII staining revealed punctate structures for Nesprin-1 in the cytoplasm of the MDA-MB-231 cells, without any prominent nuclear rim staining observed (Figure S7A). To gain further insights into Nesprin-1 localisation, the pattern of Nesprin-1 using both specII and N1ABD antibodies was examined in the non-tumorigenic MCF10A cell line. In contrast to the N1ABD antibody, the specII staining revealed prominent nuclear rim Nesprin-1 staining in the MCF10A cells (Figure S7B). The quantitative analysis showed that 74.7% of the MCF10A cells exhibited Nesprin-1 staining at the nuclear envelope, compared to only 2% of the MDA-MB-231 cells (Figure S7C). This evidence not only confirms the functionality of the specII antibody, but also highlights abnormalities in the nuclear localisation of Nesprin-1 in the MDA-MB-231 cells compared to the non-metastatic MCF10A epithelial cells. 

### 3.4. The Levels of SUN1, SUN2, Lamin A/C, and Lamin B1 Proteins Are Unaffected in AGK2-Treated MDA-MB-231 Cells Compared to Controls 

**Figure 4:**
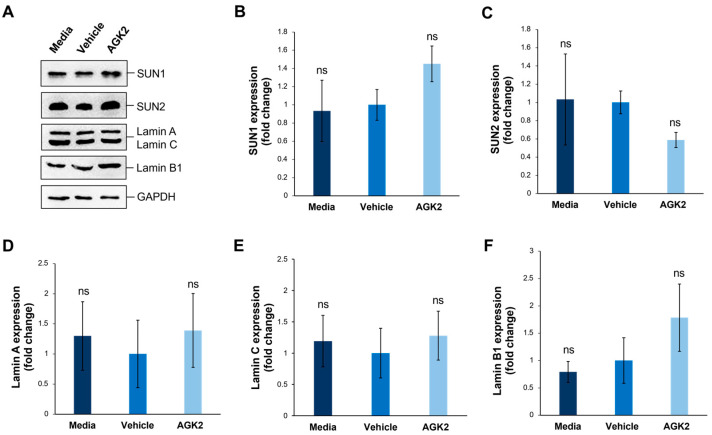
SIRT2 inhibition does not impact the expression of SUN1, SUN2, lamin A/C, and lamin B1 proteins in MDA-MB-231 cells. (**A**) Western blot analysis of SUN1, SUN2, lamin A/C, and Lamin B1 protein expression in media-, vehicle-, and AGK2-treated MDA-MB-231 cells, with GAPDH levels demonstrating equal protein loading. (**B**–**F**) Densitometric quantification of SUN1 (**B**), SUN2 (**C**), lamin A (**D**), lamin C (**E**), and lamin B1 (**F**) protein expression levels, normalised against the GAPDH loading control and presented as a mean fold change relative to the vehicle treatment. The data are presented as the mean ± SEM, n = 3. Statistical significance was assessed using a one-way ANOVA, comparing each group to the vehicle control. “ns” indicates non-significance.

Given that SUN-domain proteins and the nuclear lamina are essential for tethering Nesprins to the nuclear envelope, we further explored whether AGK2 treatment affects the expression levels of SUN1, SUN2, lamin A/C, and lamin B1 in MDA-MB-231 cells. Additionally, since the lamin A levels correlate with tissue stiffness, this analysis aimed to provide further insights into the mechanical properties of the examined cells. To investigate this, we performed Western blotting using antibodies against the aforementioned proteins, with GAPDH acting as a loading control (Figure 4A). For the SUN proteins, quantification revealed a 1.45-fold upregulation in the SUN1 levels and a 0.59-fold downregulation in the SUN2 levels in the AGK2-treated cells relative to the vehicle control, neither of which were deemed significant changes by a one-way ANOVA (Figure 4B,C). Furthermore, the AGK2-treated cells displayed no significant difference in the expression of lamin A or lamin C in comparison to the controls (Figure 4D,E). Similarly, although the lamin B1 expression increased 1.78-fold upon AGK2 treatment, this upregulation was insignificant compared to that in the vehicle-treated MDA-MB-231 cells (Figure 4F). Therefore, this further indicates that changes in the expression of the LINC complex or nuclear lamina components do not lead to alterations in the cytoskeletal organisation observed upon SIRT2 inhibition in MDA-MB-231 cells.

### 3.5. AGK2-Induced SIRT2 Inhibition Prevents Nuclear Collapse in MDA-MB-231 Cells Subjected to Osmotic Shock

**Figure 5:**
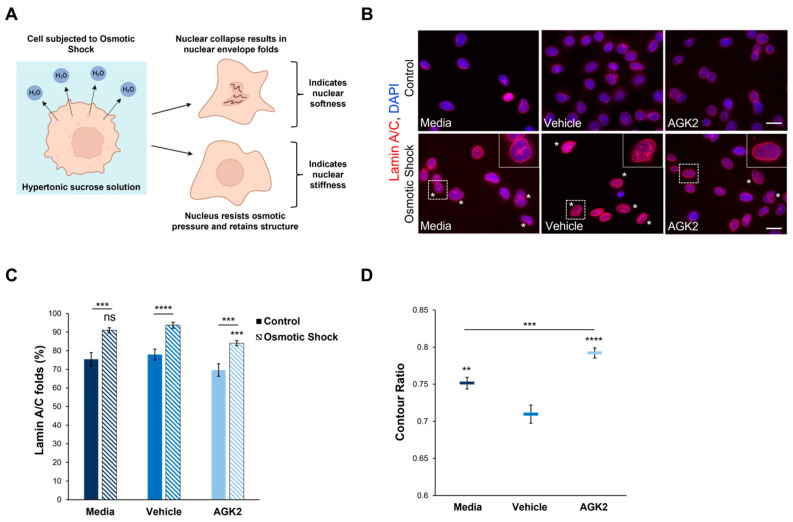
The nuclei of AGK2-treated MDA-MB-231 cells exhibit greater resistance to collapse under osmotic shock compared to controls. (**A**) A schematic demonstrating the osmotic shock assay as a method to study nuclear mechanics. Subjection to the hypertonic 640 mOsm sucrose solution results in the outward movement of water from the cell. Nuclei that undergo collapse and exhibit nuclear envelope folds suggest an inability to resist the osmotic pressure, indicating nuclear softness. In contrast, nuclei that retain their morphology under osmotic shock imply greater rigidity. (**B**) Lamin A/C immunofluorescence microscopy images of control and AGK2-treated MDA-MB-231 cells cultured under normal growth conditions (control, upper row) or subjected to osmotic shock (lower row). Lamin A/C stain (red channel) acts as a nuclear envelope marker, enabling the identification of cells exhibiting nuclear envelope folds, which are highlighted by asterisks (*). DAPI stain was used to visualise nuclei (blue channel). Insets (osmotic shock condition, lower row) are higher magnifications of the areas indicated by dashed boxes. Scale bars (lower and upper row): 20 μm. (**C**) Quantification of the percentage (%) of media-, vehicle-, and AGK2-treated cells exhibiting lamin A/C folds when cultured under control conditions or following osmotic shock; >200 cells analysed for each condition. (**D**) Quantification of the contour ratio (nuclear circularity) of control and AGK2-treated MDA-MB-231 cells following subjection to osmotic shock; >200 cells analysed per condition. The data in (**C**,**D**) are presented as the mean ± SEM. Statistical significance between cells grown under control and osmotic shock conditions for each treatment (**C**) was determined using a Student’s unpaired t-test, with statistically significant results indicated by underlined labels. Statistical analysis of media-, vehicle-, and AGK2-treated MDA-MB-231 cells subjected to osmotic shock (**C**,**D**) was conducted using a one-way ANOVA, followed by a Dunnett’s post hoc test relative to the vehicle treatment. “ns” demonstrates non-significance, while ** *p* ≤ 0.01, *** *p* ≤ 0.001, and **** *p* ≤ 0.0001 signify statistical significance.

Given the role of F-actin and vimentin in maintaining nuclear mechanics, we were interested in investigating whether the perinuclear organisation of these cytoskeletal networks modulates the nuclear stiffness of AGK2-treated MDA-MB-231 cells. To assess nuclear rigidity, the control and AGK2-treated cells were subjected to an osmotic shock assay using a 640 mOsm hypertonic sucrose solution. Exposure to the hypertonic sucrose solution causes water to move out of the cell, generating osmotic pressure, which differentially induces nuclear collapse dependent on the nucleus’s stiffness (Figure 5A). Following subjection to osmotic shock, lamin A/C immunofluorescence staining was conducted to visualise the presence of nuclear envelope folds (Figure 5B). This enabled us to identify which nuclei collapsed under the osmotic pressure (marked by the asterisks in Figure 5B) and which resisted the osmotic pressure, retaining their original morphology. To ensure the sucrose concentration was sufficient to induce effects in MDA-MB-231 nuclei, the percentage of cells exhibiting nuclear envelope folds under control conditions (Figure 5B, upper row) and osmotic shock conditions (Figure 5B, lower row) was quantified. This analysis revealed that subjection to the osmotic shock assay significantly increased the percentage of lamin A/C folds by 15% in the media-treated cells compared to their untreated control (isotonic condition) counterparts (Figure 5C). Thus, this demonstrates that the osmotic stress in MDA-MB-231 cells generated sufficient pressure to affect the nuclear morphology.

Subsequently, the percentage of nuclear envelope folds in the control and AGK2-treated MDA-MB-231 cells after osmotic shock treatment was examined. This analysis revealed that 90.9% of the media-treated and 93.7% of the vehicle-treated cells exhibited lamin A/C folds (Figure 5C), as demonstrated by the insets in Figure 5B. Thus, this indicates that most of the control MDA-MB-231 cells were unable to resist the osmotic pressure. In contrast, the AGK2-treated MDA-MB-231 cells retained their nuclear structure more frequently, as highlighted by the inset in Figure 5B, with quantification confirming that the AGK2 treatment significantly reduced the percentage of cells exhibiting lamin A/C folds to 84% (Figure 5C). This is further supported by quantifying the contour ratio of nuclei following osmotic shock, which found that the AGK2 treatment significantly increased the nuclear circularity compared to in the media-treated and vehicle-treated cells (Figure 5D). Together, these results suggest that the nuclei of AGK2-treated MDA-MB-231 cells are less likely to undergo collapse upon subjection to osmotic shock. 

### 3.6. AGK2-Treated MDA-MB-231 Cells Display Migratory and Polarity Defects in Response to Wounding

**Figure 6:**
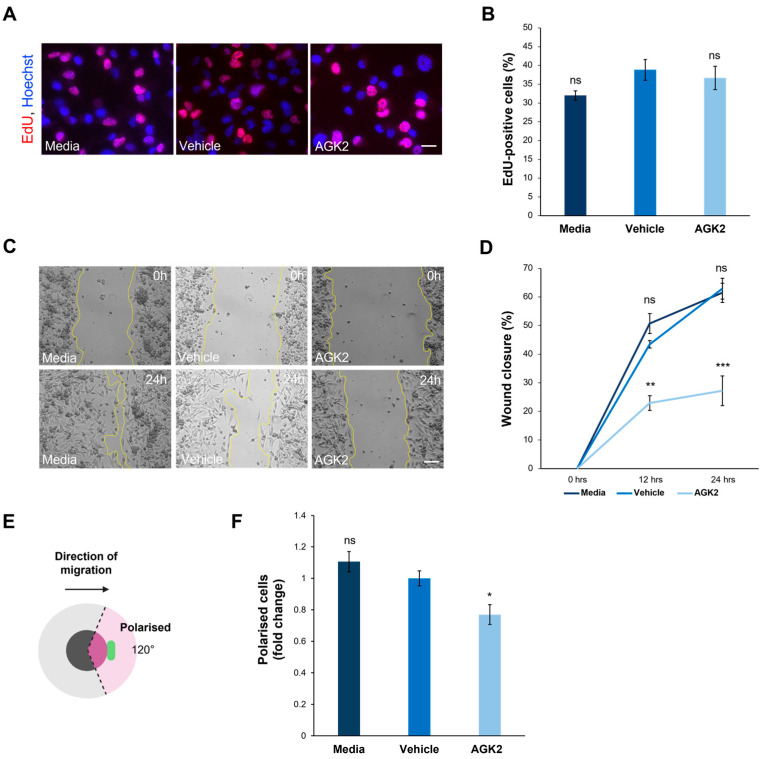
AGK2 treatment impedes the migration and polarisation of MDA-MB-231 cells in a 2D environment. (**A**) Fluorescence microscopy images of media-, vehicle-, and AGK2-treated MDA-MB-231 cells subjected to the EdU proliferation assay. EdU-positive cells are indicated by the red channel, while Hoechst labelling indicates nuclear staining on the blue channel. Scale bar: 20 μm. (**B**) Quantification of the percentage (%) of EdU-positive cells for each treatment; >1000 cells analysed per condition. (**C**) Phase contrast images of the scratch-wound assay at 0 h (upper row) and 24 h (lower row) post-wounding for control and AGK2-treated cells. Scale bar: 100 μm. (**D**) Quantification of the percentage (%) wound closure at 0, 12, and 24 h of the cell wounding experiment for each treatment, n = 3. (**E**) A schematic depicting the parameters by which cells were characterised as polarised in the scratch-wound assay. Cells were classified as polarised when the Golgi organelle was situated within the 120° radius (pink-shaded area) facing the wound edge. (**F**) Quantification of the number of polarised cells for each treatment, displayed as a fold change relative to the vehicle control; >300 cells analysed per condition. The data in (**B**,**D**,**F**) are presented as the mean ± SEM, and statistical significance was assessed using a one-way ANOVA, followed by a Dunnett’s post hoc test comparing the data to the vehicle treatment. “ns” denotes non-significant results, while * *p* ≤ 0.05, ** *p* ≤ 0.01, and *** *p* ≤ 0.001 indicates statistical significance.

To further understand the impact of SIRT2 inhibition on the biology of the MDA-MB-231 cells, additional experiments were conducted focusing on the effect of the AGK2 treatment on their proliferative and migratory capacity. The former was determined using an EdU assay, followed by fluorescence microscopy to quantify the EdU-positive cells (Figure 6A). This analysis demonstrated that SIRT2 inhibition had no significant effects on the proliferative ability of the MDA-MB-231 cells, with 38.8% of the vehicle-treated and 36.6% of the AGK2-treated cells displaying EdU-positive staining (Figure 6B). The immunofluorescence staining for Ki67, a widely used marker of proliferation in human tumour cells, further confirmed this finding by revealing no significant differences between the percentage of Ki67-positive cells in the control and AGK2-treated conditions (Figure S8A,B).

With the AGK2-treated MDA-MB-231 cells exhibiting more prominent perinuclear cytoskeletal organisations, the next step was to explore whether this affected cell migration in a 2D scratch-wound assay (Figure 6C). The quantification of the percentage wound closure at 0h, 12 h, and 24 h showed that the AGK2 treatment significantly impeded the wound healing capacity of the MDA-MB-231 cells, displaying only 27.2% wound closure after 24 h compared to 62.9% in the vehicle control (Figure 6D). Furthermore, the ability of the Golgi apparatus to polarise towards the wound edge in response to the scratch-wounding was examined (Figure 6E). This highlighted that significantly fewer AGK2-treated cells polarised towards the scratch in comparison to the control conditions, suggesting that SIRT2 inhibition induces polarity defects in MDA-MB-231 cells (Figure 6F).

### 3.7. SIRT2 Inhibition Dampens the Invasive Capacity of MDA-MB-231 Cells Through Space-Restrictive 3D In Vitro Environments

**Figure 7:**
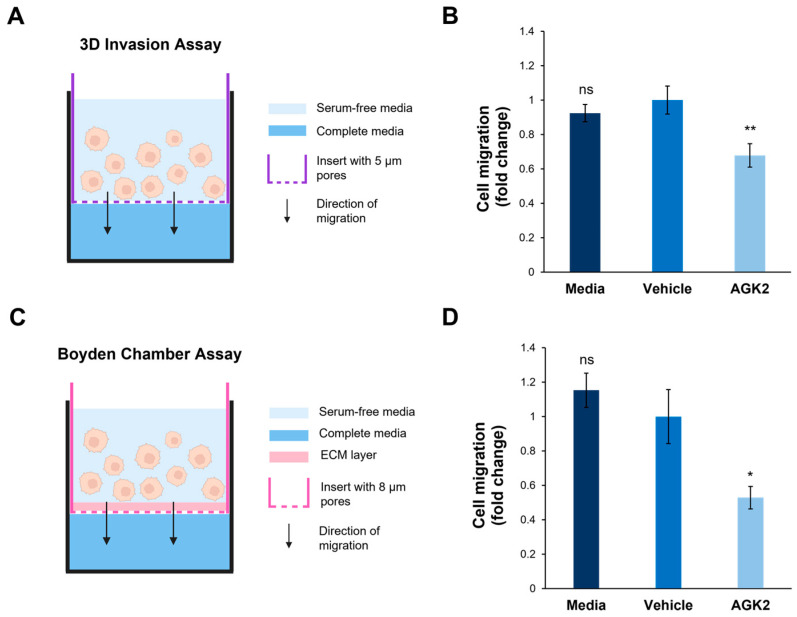
SIRT2 inhibition reduces the migration of MDA-MB-231 cells through space-restricted 3D environments. (**A**) A schematic demonstrating the setup of the 3D space-restrictive invasion assay, where an insert with a porous membrane (indicated by a purple dashed line, pore diameter: 5 μm) acts as a restrictive barrier for cells migrating from an area of serum-free media (light blue shading) to complete media (dark blue shading). (**B**) Quantification of media-, vehicle-, and AGK2-treated cell migration within the 3D space-restrictive invasion assay, presented as a fold change relative to the vehicle treatment. (**C**) A schematic showing the setup of the well-established Boyden Chamber assay, in which the restrictive barrier between the serum-free media (light blue shading) and complete media (dark blue shading) consists of a layer of extracellular matrix (ECM, pink shading) as well as a porous membrane (indicated by a pink dashed line, pore diameter: 8 μm). (**D**) Quantification of media-, vehicle-, and AGK2-treated cell migration within the Boyden Chamber assay, presented as a fold change relative to the vehicle treatment. The data in (**B**,**D**) are shown as the mean ± SEM, and statistical significance was determined using a one-way ANOVA followed by a Dunnett’s post hoc test relative to the vehicle treatment. “ns” indicates non-significant results, while * *p* ≤ 0.05 and ** *p* ≤ 0.01 indicate statistical significance.

In addition to exploring cell migration in a 2D environment, the impact of SIRT2 inhibition on the invasive capacity of the MDA-MB-231 cells in a 3D environment was assessed. This was particularly relevant considering the results of the osmotic shock assay, which suggested that the AGK2-treated cells displayed alterations in the biomechanical properties of their nuclei. To achieve this, a space-restrictive invasion assay was first performed using a 5 μm porous membrane (Figure 7A). This revealed that the AGK2 treatment significantly inhibited the migratory capacity of the MDA-MB-231 cells compared to the controls (Figure 7B). To more comprehensively understand MDA-MB-231 invasion upon SIRT2 inhibition, a Boyden Chamber assay, containing an extracellular matrix (ECM) layer above an 8 μm porous membrane, was then conducted (Figure 7C). In this experimental setup, the AGK2-treated MDA-MB-231 cells also displayed significantly impeded invasion compared to vehicle-treated cells (Figure 7D). Thus, these results imply that SIRT2 inhibition significantly reduces the movement of MDA-MB-231 cells through confined in vitro environments, indicating the potential for AGK2 treatment to dampen MDA-MB-231 cells’ enhanced migratory capacity.

### 3.8. In HCC38 Cells with Low SIRT2 Expression, AGK2 Treatment Has Minimal Effects on the Acetylated α-Tubulin Cytoskeletal Organisation and Migration 

**Figure 8:**
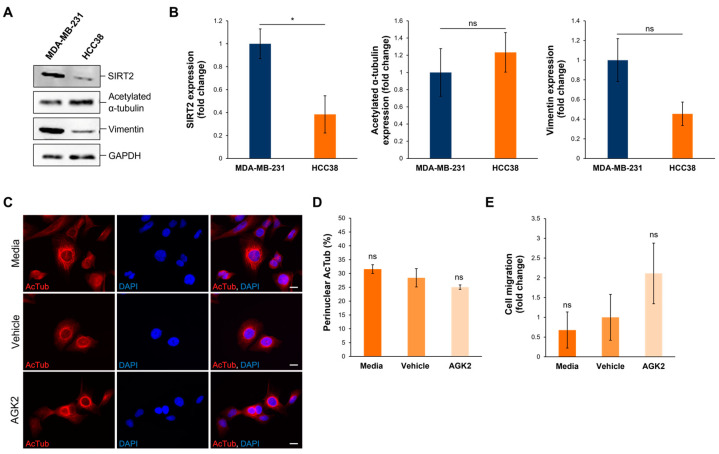
HCC38 cells display reduced SIRT2 expression compared to MDA-MB-231 cells and show no change in migratory capacity upon AGK2 treatment. (**A**) Western blot analysis showing SIRT2, acetylated α-tubulin, and vimentin protein expression levels in MDA-MB-231 cells compared to HCC38 cells. GAPDH expression is used to demonstrate equal protein loading. (**B**) Densitometric quantification of SIRT2, acetylated α-tubulin, and vimentin protein expression in MDA-MB-231 and HCC38 cells, normalised against the GAPDH loading control and presented as an average fold change relative to the protein expression in MDA-MB-231 cells. The data are presented as the mean ± SEM, n = 3. (**C**) Immunofluorescence microscopy images of acetylated α-tubulin (AcTub; red channel) organisation in media-, vehicle-, and AGK2-treated HCC38 cells. Nuclei are visualised using a DAPI counterstain (blue channel). Scale bar: 10 μm. (**D**) Quantification of the percentage (%) of control and AGK2-treated HCC38 cells exhibiting prominent perinuclear acetylated α-tubulin rings; >300 cells analysed per condition. (**E**) Quantification of media-, vehicle-, and AGK2-treated HCC38 cell migration within the 3D space-restrictive invasion assay (5 μm diameter pores), presented as a fold change relative to the vehicle treatment. Statistical significance in panel (**B**) was determined by a Student’s unpaired t-test, and in panels (**D**,**E**) using a one-way ANOVA, comparing each group to the vehicle control. “ns” indicates non-significance, while * *p* ≤ 0.05 denotes statistical significance.

To further validate the data obtained from examining SIRT2 inhibition in MDA-MB-231 cells, the Human Protein Atlas (www.proteinatlas.org, accessed on date: 13 November 2024) was consulted to identify HCC38 cells, a triple-negative breast cancer (TNBC) cell line exhibiting low SIRT2 expression at the transcriptional level. According to the Human Protein Atlas database, MDA-MB-231 cells express 2.47 times more SIRT2 transcripts than HCC38 cells do. This difference in SIRT2 expression was further confirmed at the protein level, with Western blotting demonstrating that the MDA-MB-231 cells exhibited a significant 2.57-fold increase in SIRT2 protein compared to the HCC38 cells (Figure 8A,B). In contrast to SIRT2, however, the HCC38 cells showed no significant differences in the expression of acetylated α-tubulin and vimentin in comparison to the MDA-MB-231 cells (Figure 8A,B). Thus, as HCC38 cells possess intrinsically lower levels of SIRT2 protein, this cell line provides a control for AGK2-treated MDA-MB-231 cells. 

Subsequently, HCC38 cells were subjected to 24 h treatment with 5 μM AGK2 and the effect of SIRT2 inhibition on the cytoskeletal organisation and migratory capacity was determined. Immunofluorescence staining for acetylated α-tubulin revealed that media-, vehicle-, and AGK2-treated HCC38 cells all exhibit prominent perinuclear organisations of this microtubule component (Figure 8C). Thus, in contrast to the MDA-MB-231 cells, the quantification confirmed that the AGK2 treatment had no significant impact on the percentage of HCC38 cells displaying perinuclear acetylated α-tubulin rings (Figure 8D). Furthermore, the AGK2 treatment did not significantly alter the invasiveness of the HCC38 cells in the space-restrictive 3D migration assay. Together, this demonstrates that SIRT2 inhibition is less effective in HCC38 cells with low SIRT2 expression, highlighting that the effects of AGK2 treatment in MDA-MB-231 cells are specifically due to reduced SIRT2 activity. 

## 4. Discussion

This study demonstrated that a 24 h treatment with 5 μM AGK2 enhanced the perinuclear organisation of acetylated α-tubulin, vimentin, and F-actin cytoskeletal networks, while the LINC complex composition remained unchanged. In addition, the AGK2 treatment increased the resistance of the MDA-MB-231 cells to nuclear collapse under osmotic shock and impeded their invasion through space-restrictive in vitro environments. Together, this evidence suggests that AGK2-induced SIRT2 inhibition likely modulates the nuclear rigidity of MDA-MB-231 cells via the integrated interactions between the nucleus and the perinuclear cytoskeleton.

Whilst the Western blotting detected no significant change in the SIRT2 protein levels in the AGK2-treated MDA-MB-231 cells, this is consistent with AGK2 acting as a competitive inhibitor of SIRT2 by blocking the NAD^+^-binding site [60]. Furthermore, the inability of the AGK2 treatment to impact the expression of SIRT2 downstream targets is supported by evidence showing that higher concentrations of AGK2 are required to affect the protein levels of acetylated α-tubulin and acetylated histone 4 [66,67,68]. Nevertheless, previous work has found the organisation of acetylated α-tubulin to be a better indicator of AGK2-mediated SIRT2 inhibition than the acetylated α-tubulin protein levels themselves [47]. Consequently, the enhanced perinuclear organisation of acetylated α-tubulin in the AGK2-treated MDA-MB-231 cells demonstrated that a 24 h 5 μM AGK2 treatment suppresses SIRT2 enzymatic activity by increasing the levels of acetylated α-tubulin locally, as the effect is restricted to the perinuclear region. This is further confirmed by the low-SIRT2-expressing HCC38 cells displaying pronounced perinuclear acetylated α-tubulin rings under both the control and AGK2-treated conditions. Ultimately, this finding supports the notion that the perinuclear microtubule network is a primary target of SIRT2 and aligns with evidence that AGK2 treatment leads to the hyperacetylation of perinuclear α-tubulin in HeLaS3 cells [62]. 

In agreement with previous evidence that MDA-MB-231 cells have reduced keratin 8/18 expression compared to that in MCF10A cells and display vimentin as their most prominent intermediate filament network [69,70,71], we did not detect an extensive keratin 8/18 network in these cells. Therefore, we were unable to evaluate the previous findings of perinuclear keratin 8 accumulation following AGK2 treatment in HepG2 cells [58]. Nevertheless, the increase in perinuclear vimentin and F-actin in the AGK2-treated MDA-MB-231 cells provides novel insights into the ability of SIRT2 inhibition to reorganise these cytoskeletal networks. Unlike acetylated α-tubulin, however, vimentin and F-actin have not been reported as direct targets of SIRT2. Thus, the perinuclear organisations of these cytoskeletal networks most likely result from an indirect mechanism induced by changes downstream of SIRT2. Although the AGK2 treatment did not alter the acetylated histone 4 levels and nuclear size, we cannot rule out that changes in chromatin plasticity also contribute to the perinuclear cytoskeletal alterations.

Whilst Nesprin-1 giant and Nesprin-2 giant are known to tether F-actin to the nuclear envelope and mediate the formation of an F-actin cage around the nucleus [27,72], the AGK2 treatment did not significantly alter the expression nor localisation of Nesprin-1 and -2 in the MDA-MB-231 cells. Thus, this suggests that they are likely not responsible for the perinuclear cytoskeletal reorganisation. However, we cannot exclude the possibility that AGK2 treatment enhances the binding of the altered cytoskeleton to Nesprin proteins based on previous evidence identifying Nesprin-1 as a SIRT2 protein binding partner [73]. Despite this, even though the AGK2 treatment led to a modest increase in Nesprin-1 giant expression, this protein is unlikely to contribute to the perinuclear F-actin organisation, considering that two different Nesprin-1 antibodies (N1ABD and specII) established a lack of nuclear envelope-localised Nesprin-1 in the MDA-MB-231 cells. Although the MCF10A cells also showed predominant N1ABD staining in the cytoplasm, the specII antibody exhibited a strong nuclear rim pattern in this non-invasive breast epithelial cell line. This demonstrates that, in contrast to MDA-MB-231 cells, MCF10A cells retain higher levels of Nesprin-1 at the nuclear envelope. This previously unreported result highlights a disparity in Nesprin-1 localisation between MCF10A and MDA-MB-231 cells, which may contribute to the invasiveness of the triple-negative breast cancer cell line. With the levels of SUN1, SUN2, lamin A/C, and lamin B1 also remaining unchanged in the AGK2-treated MDA-MB-231 cells, our results ultimately suggest that alterations in the LINC complex and nuclear lamina composition are unlikely to be directly involved in the perinuclear cytoskeletal organisation observed upon SIRT2 inhibition. This is also supported by the AGK2-treated MDA-MB-231 cells retaining their abnormal nuclear morphology, further indicating that the LINC complex and nuclear lamina composition remain unchanged [26,37,74]. Alternatively, it is possible that the perinuclear acetylated α-tubulin rings recruit vimentin to the perinuclear region. Extensive literature demonstrates the ability of microtubules and vimentin to interact indirectly via the microtubule motor proteins dynein and kinesin or through the cytolinker plectin, with more recent evidence also highlighting a direct interaction between the two cytoskeletal networks [75,76,77,78]. Similarly, as vimentin can bind F-actin directly through its C-terminal ‘tail’ domain [79,80] or indirectly via plectin [81], this results in interpenetrating networks that function synergistically [82,83]. Consequently, it is feasible that the perinuclear vimentin networks may be responsible for F-actin localising at the nuclear envelope of a small subset of AGK2-treated cells. 

Furthermore, the AGK2 treatment reduced the incidence of nuclear collapse in the MDA-MB-231 cells under osmotic pressure. This suggests that AGK2-induced SIRT2 inhibition increases the rigidity of MDA-MB-231 nuclei, highlighting a previously unreported link between SIRT2 and nuclear mechanics. This is based on evidence showing that the nuclei of MCF10A cells exhibit greater stiffness compared to that of MDA-MB-231 nuclei [15,84,85], which leads to their ability to maintain nuclear structure under osmotic shock, while MDA-MB-231 nuclei undergo significant deformation [71]. Previous studies implicate tubulin acetylation and the presence of both vimentin and F-actin in increasing cellular stiffness [83,86], with the loss of the latter two networks known to result in nuclear and perinuclear softening [15,42]. Thus, we propose that the prominent perinuclear cytoskeletal networks present in AGK2-treated cells are involved in promoting the stiffening of MDA-MB-231 nuclei. However, as the perinuclear vimentin rings were observed much more frequently than the perinuclear F-actin rings, they are likely to be the dominant contributor to the observed change in nuclear mechanics upon AGK2 treatment. This is supported by the ability of vimentin to resist stronger mechanical deformation than the actin and microtubule networks, with further evidence demonstrating the ability of a perinuclear vimentin cage to protect the nucleus from undergoing major deformation upon migration through confined spaces [42,87,88].

Building on this, our study extended the current knowledge on the ability of SIRT2 inhibition to impact cancer cell migration. Firstly, we showed that AGK2 treatment impedes MDA-MB-231 migration in a 2D scratch-wound assay. Importantly, as the AGK2 treatment had no impact on the proliferative capacity of the MDA-MB-231 cells, this supports the theory that SIRT2 inhibition was specifically responsible for the diminished migratory capacity of the AGK2-treated MDA-MB-231 cells. Moreover, this finding is coherent with literature that has reported similar findings in HeLa and osteosarcoma cells [65,89]. Tian et al. (2022) attributed the decrease in osteosarcoma cell migration to a reduction in the deacetylation of SIRT2’s downstream target, Snail. This resulted in increased Snail degradation and, ultimately, the suppression of the epithelial-to-mesenchymal transition (EMT) and mesenchymal protein expression. However, as the vimentin expression remained unchanged in the AGK2-treated MDA-MB-231 cells, it is unlikely that the AGK2-induced SIRT2 inhibition was sufficient to downregulate the EMT. Nevertheless, alternative studies have implicated different SIRT2 downstream targets in the regulation of gastric cancer and hepatocellular carcinoma migration [63,64], supporting that SIRT2 inhibition may modulate the migratory capacity of different cancer types via diverse mechanisms. Consequently, as previous studies clearly show that the Golgi must become repositioned between the nucleus and leading edge for directional cell migration to occur [90,91,92,93], we hypothesise that the polarity defects exhibited by AGK2-treated MDA-MB-231 cells strongly contribute to their inability to heal the scratch-wound. Importantly, the ability of the AGK2 treatment to impact cell polarity is also consistent with previous evidence demonstrating that SIRT2 knockdown leads to abnormal Golgi polarisation in neurons [94]. Additionally, microtubules, vimentin, and F-actin are known to play key roles in nuclear positioning during cell polarisation due to their ability to interact with the LINC complex [95,96,97,98,99]. More specifically, transmembrane actin-associated nuclear (TAN) lines facilitate retrograde nuclear movement during polarisation [100], whilst tubulin and vimentin are essential for the repositioning of the centrosome towards the wound edge [101,102,103]. Thus, it is possible that the prominent perinuclear acetylated α-tubulin, vimentin, and F-actin networks prevent the cytoskeletal remodelling required for cell polarisation, consequently impeding the 2D migration of MDA-MB-231 cells towards the wound edge. 

With 3D cell migration particularly reliant on the actin cytoskeleton forming pseudopodial protrusions and generating contractile forces [104], the reorganisation of F-actin around the nucleus may impair the migration of AGK2-treated MDA-MB-231 cells within the 3D invasion and Boyden Chamber assays. Moreover, as the presence of a perinuclear vimentin cage was found to impede the invasion of mouse embryonic fibroblasts (mEFs) within a Transwell assay [42,105], the perinuclear vimentin rings exhibited by the AGK2-treated MDA-MB-231 cells likely contribute to their reduced migratory capacity in the 3D environment. Ultimately, the inability of the AGK2 treatment to impede the 3D migration of the low-SIRT2-expressing HCC38 cells in space-restrictive settings indicates that the dampened invasiveness of MDA-MB-231 cells specifically results from SIRT2 inhibition. Importantly, this result is consistent with SIRT2 inhibition reducing the migratory and invasive capacity of gastric cancer and osteosarcoma cells in Transwell assays both with and without Matrigel [64,65]. Moreover, these publications demonstrate the ability of SIRT2 inhibition to impede the metastasis of xenograft tumours [64,65], highlighting the potential for AGK2 treatment to achieve a similar outcome. However, considering that these studies inhibit SIRT2 activity using a knockdown approach, further investigation is needed to determine whether the pharmacological inhibition of SIRT2, using AGK2, is sufficient to restrict MDA-MB-231 metastases in vivo. 

Finally, with nuclear rigidity regarded as a rate-limiting factor of cell migration through confined environments [13,106], the dampened ability of AGK2-treated MDA-MB-231 cells to migrate through space-restrictive pores provides additional evidence that SIRT2 inhibition induces nuclear stiffening. Previously published work has clearly established that enhancing the nuclear stiffness of cells, by inducing lamin A overexpression, diminishes their invasiveness [107,108]. Therefore, this implies that the increased nuclear rigidity of AGK2-treated MDA-MB-231 cells contributes to the underlying mechanism leading to their reduced invasive capacity in the 3D environment. In turn, this further supports our hypothesis that the perinuclear cytoskeleton observed upon AGK2 treatment enhances the nuclear stiffness of MDA-MB-231 cells.

## 5. Conclusions

This study demonstrated the ability of SIRT2 inhibition to modulate the nuclear mechanics of MDA-MB-231 cells. This is important considering that TNBC cells display softer nuclei than healthy breast epithelia, leading to their enhanced migratory capacity within the 3D environment. Our results highlight that, unlike the MCF10A cells, the MDA-MB-231 cells lacked Nesprin-1 localisation at the nuclear envelope, which likely contributed to their reduced nuclear stiffness. Whilst the low-SIRT2-expressing HCC38 cells were unaffected by the AGK2 treatment, SIRT2 inhibition in the high-SIRT2-expressing MDA-MB-231 cells induced significant effects on the cytoskeletal organisation and migration in both the 2D scratch-wound and 3D space-restrictive in vitro environments. Since the composition and expression levels of the LINC complex and nuclear lamina proteins remained unchanged in the AGK2-treated MDA-MB-231 cells, the impact of SIRT2 inhibition on nuclear mechanobiology is attributed to the pronounced perinuclear cytoskeleton. Moreover, our data suggest that the increased nuclear stiffness and polarity defects displayed by the AGK2-treated MDA-MB-231 cells are responsible for their dampened migratory and invasive capacity. Consequently, further research into the molecular associations between the cytoskeleton and nucleus following SIRT2 inhibition could yield valuable insights for reducing cancer cell invasiveness. 

## Data Availability

The raw data supporting the conclusions of this article will be made available by the authors on request.

## Supplementary Materials

The following supporting information can be downloaded at https://www.mdpi.com/article/10.3390/cells13232023/s1: Figure S1: Examination of cell viability in MDA-MB-231 cells following subjection to a range of AGK2 concentrations using an MTT assay; Figure S2: InstantBlue Coomassie staining and the analysis of ubiquitously expressed proteins confirm that MDA-MB-231 lysates contain equal amounts of cellular protein; Figure S3: The expression levels of SIRT2, SIRT2 downstream targets, and vimentin are unchanged in AGK2-treated MDA-MB-231 cells; Figure S4: MDA-MB-231 cells lack an extensive keratin 8/18 network, which is unaffected by AGK2 treatment; Figure S5: A small population of AGK2-treated MDA-MB-231 cells exhibit a perinuclear F-actin ring; Figure S6: AGK2-induced SIRT2 inhibition has no impact on the nuclear morphology of MDA-MB-231 cells; Figure S7: The localisation of Nesprin-1 differs between MDA-MB-231 and MCF10A cells; Figure S8: AGK2 treatment does not affect the proliferative capacity of MDA-MB-231 cells; Table S1: Primary antibodies for Western blotting; Table S2: Secondary antibodies for Western blotting; Table S3: Primary antibodies for immunofluorescence staining; Table S4: Secondary antibodies/fluorescent stains for immunofluorescence staining [109,110,111,112].
