# Supplementary material for: SIRT2 Inhibition by AGK2 Promotes Perinuclear Cytoskeletal Organisation and Reduces Invasiveness of MDA-MB-231 Triple-Negative Breast Cancer Cells in Confined In Vitro Models
Source: Cells. 2024 Dec 7;13(23):2023. doi: 10.3390/cells13232023 (PMC11639776; doi:10.3390/cells13232023)
Supplement: Supplementary file 1 [file cells-13-02023-s001.zip › cells-3341677-supplementary.pdf]

Supplemental Material

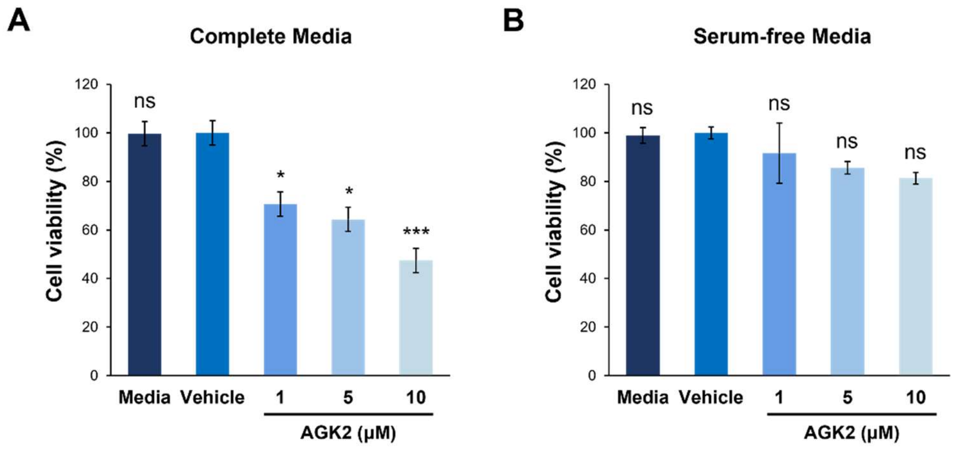

Supplement Figure 1: Examination of cell viability in MDA-MB-231 cells subjected to a range of AGK2 concentrations using an MTT assay. (**A-B**) MTT assay results for the treatment of MDA-MB-231 cells with 1 μM, 5 μM, and 10 μM AGK2 in complete media (**A**) and serum-free media (**B**). The data is presented as percentage (%) of cell viability. The data indicate the mean ± SEM, n = 3. Statistical significance was confirmed using a one-way ANOVA, followed by Dunnett's post-hoc test comparing results to the vehicle control. "ns" indicates non-significance, while $*p \le 0.05$ and $***p \le 0.001$ demonstrate statistical significance.

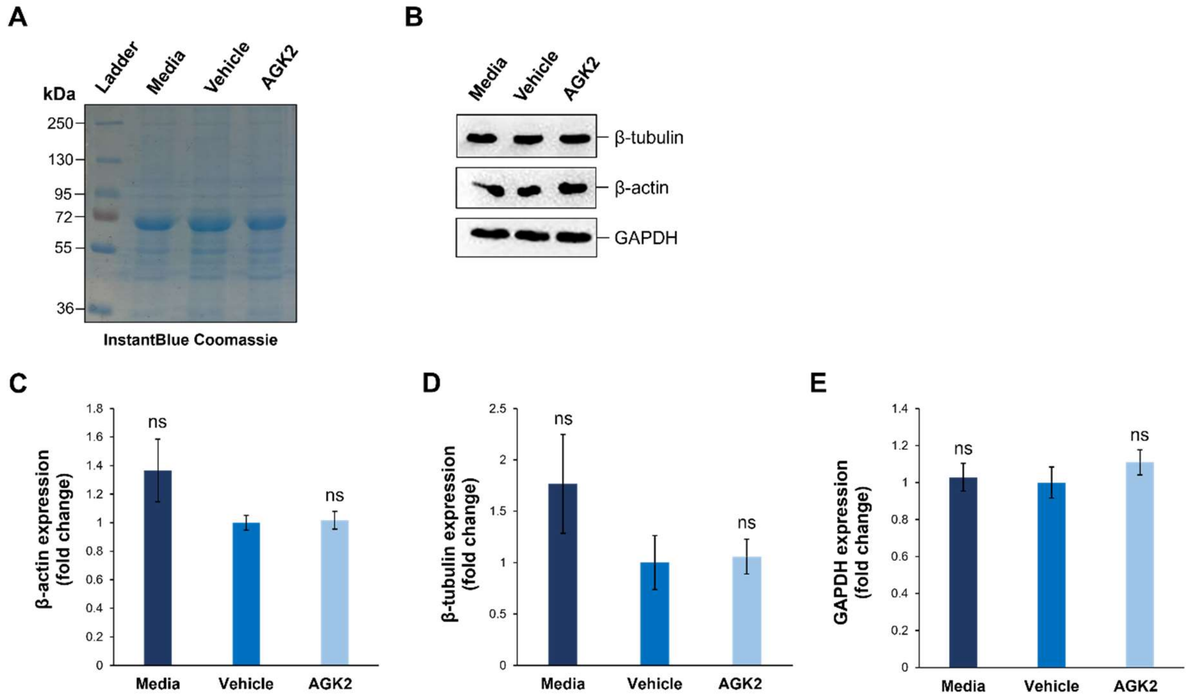

Supplement Figure 2: InstantBlue Coomassie staining and the analysis of ubiquitously expressed proteins confirm that MDA-MB-231 lysates contain equal amounts of cellular protein.

(**A**) InstantBlue Coomassie stain of the polyacrylamide gel following SDS-PAGE of MDA-MB-231 cell lysates treated with media, vehicle, or 5 μM AGK2. (**B**) Western blot analysis of the ubiquitously expressed proteins, β-tubulin, β-actin, and GAPDH, in media-, vehicle-, and AGK2-treated MDA-MB-231 cells. (**C-E**) Densitometric quantification of β-actin (**C**), β-tubulin (**D**), and GAPDH (**E**) expression levels, with (**C-D**) normalised against the GAPDH loading control and (**C-E**) presented as an average fold change relative to the vehicle treatment ± SEM, n = 3. Statistical analysis was conducted using a one-way ANOVA. "ns" denotes no significant difference.

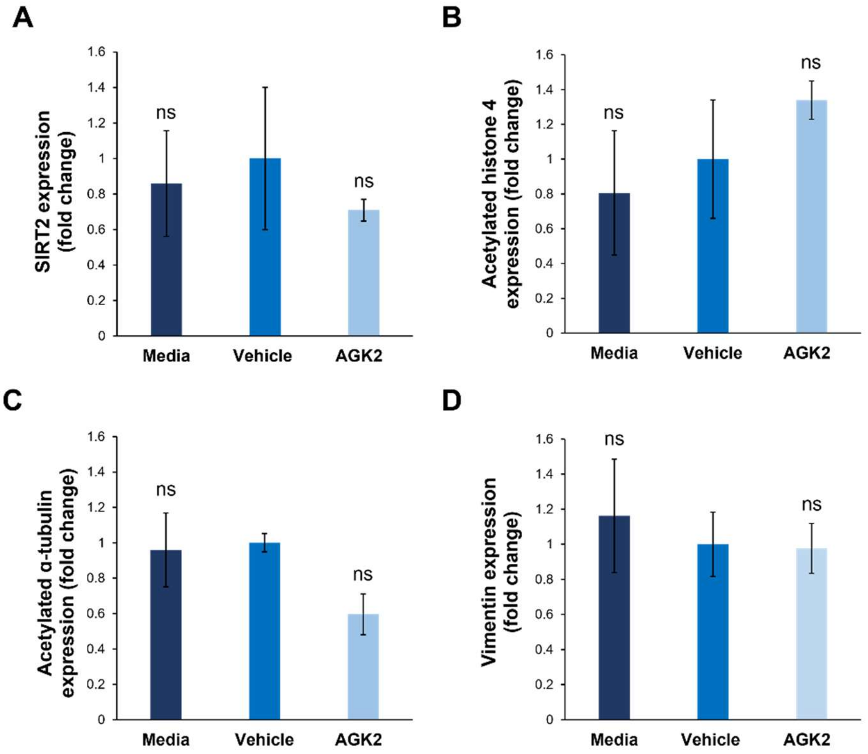

Supplement Figure 3: The expression levels of SIRT2, SIRT2 downstream targets, and vimentin are unchanged in AGK2-treated MDA-MB-231 cells. (**A-D**) Quantitative examination of SIRT2 (**A**), acetylated histone 4 (**B**), acetylated α-tubulin (**C**), and vimentin (**D**) protein levels in control and AGK2-treated MDA-MB-231 cells following Western blot analysis (shown in Figure 1A). The data was normalised against the GAPDH loading control and presented as a mean fold change in comparison to the vehicle treatment ± SEM, n = 3. Statistical significance was assessed with a one-way ANOVA, comparing each treatment group to the vehicle control. "ns" indicates no significance.

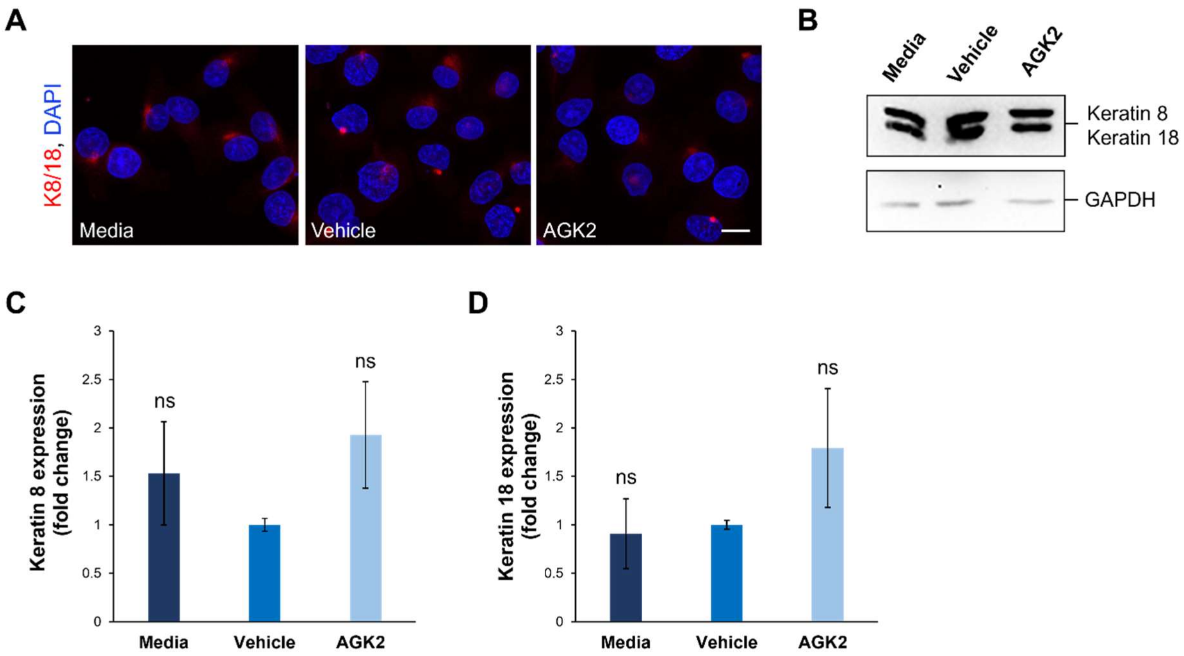

Supplement Figure 4: MDA-MB-231 cells lack an extensive keratin 8/18 network, which is unaffected by AGK2 treatment. (**A**) Representative confocal microscopy images of keratin 8/18 immunofluorescence staining in media-, vehicle-, and AGK2-treated MDA-MB-231 cells. Nuclei are labelled using a DAPI counterstain. Scale bar: 10 µm. (**B**) Immunoblot analysis of keratin 8 and keratin 18 expression in control and AGK2-treated MDA-MB-231 cells, with GAPDH used as a loading control to confirm equal protein loading. (**C-D**) Quantification of keratin 8 (**C**) and keratin 18 (**D**) protein expression levels in MDA-MB-231 cells. The data represent the mean fold change relative to the vehicle control ± SEM, n = 3. Statistical significance was determined using a one-way ANOVA to compare each treatment group to the vehicle control. "ns" denotes no significant difference.

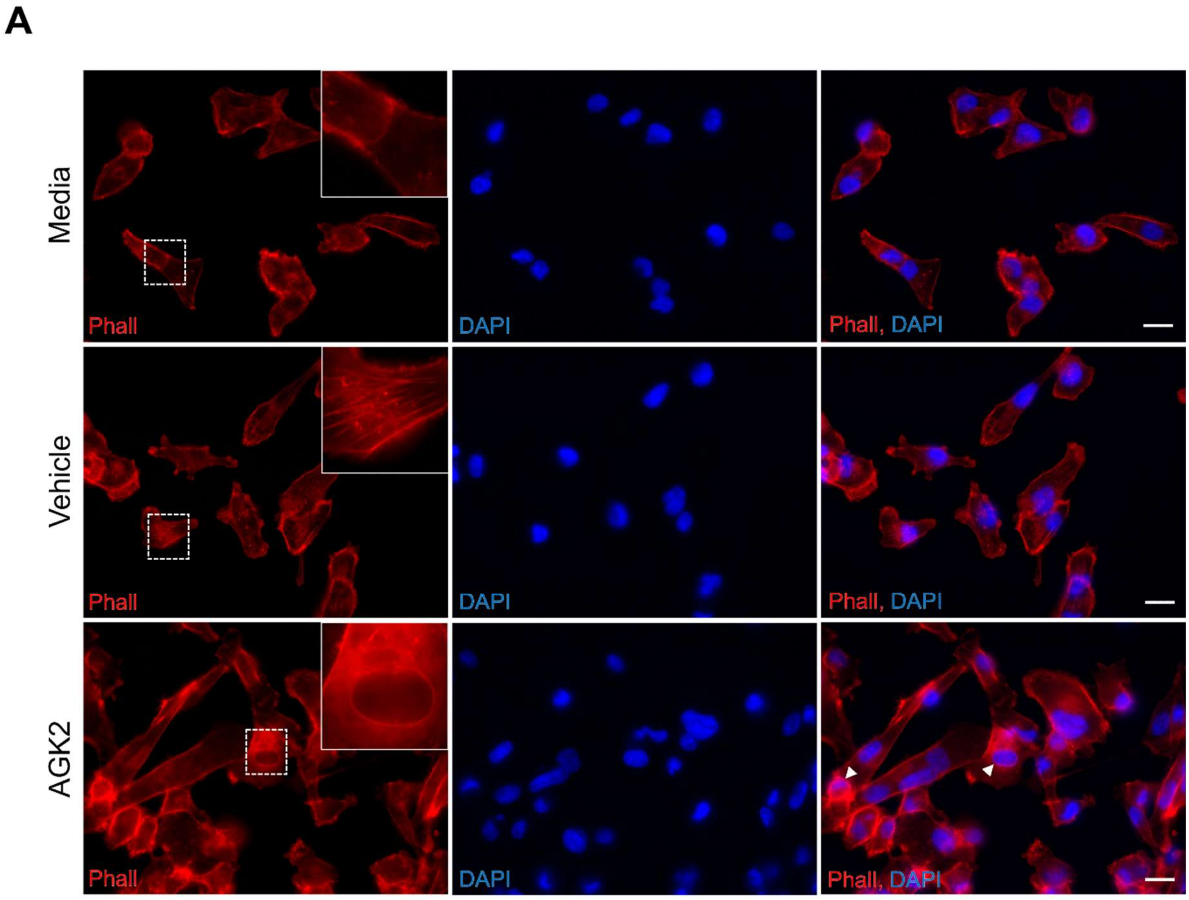

Supplement Figure 5: A small population of AGK2-treated MDA-MB-231 cells exhibit a perinuclear F-actin ring. (**A**) Fluorescence microscopy images of media-, vehicle-, and AGK2-treated MDA-MB-231 cells stained with TRITC-phalloidin (labelling F-actin) and DAPI counterstain (labelling nuclei). Insets (which show only the red channel) are higher magnifications of the dashed boxed areas. Arrowheads indicate the presence of perinuclear F-actin rings. Scale bar: 20 μm.

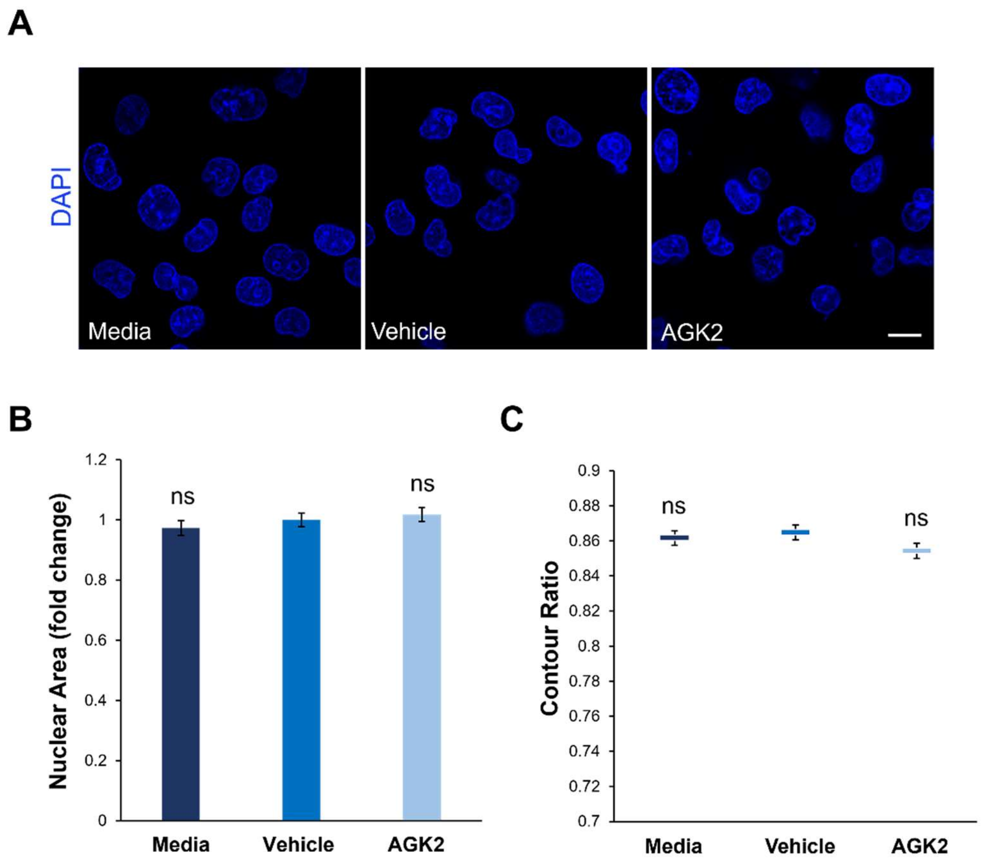

Supplement Figure 6: AGK2-induced SIRT2 inhibition has no impact on the nuclear morphology of MDA-MB-231 cells. (**A**) Confocal microscopy images of DAPI-stained MDA-MB-231 nuclei under control and AGK2-treated conditions. Scale bar: 10 µm. (**B**) Quantification of the nuclear area of media-treated, vehicle-treated, and AGK2-treated MDA-MB-231 cells, presented as a fold change relative to the vehicle control; 200 cells analysed per condition. The data indicate the mean ± SEM. (**C**) Quantification of the contour ratio (nuclear circularity) of control and AGK2-treated cells; 200 cells analysed per condition. Error bars represent ± SEM. Statistical analysis was conducted using a one-way ANOVA to compare each treatment group to the vehicle control, with “ns” indicating non-significance.

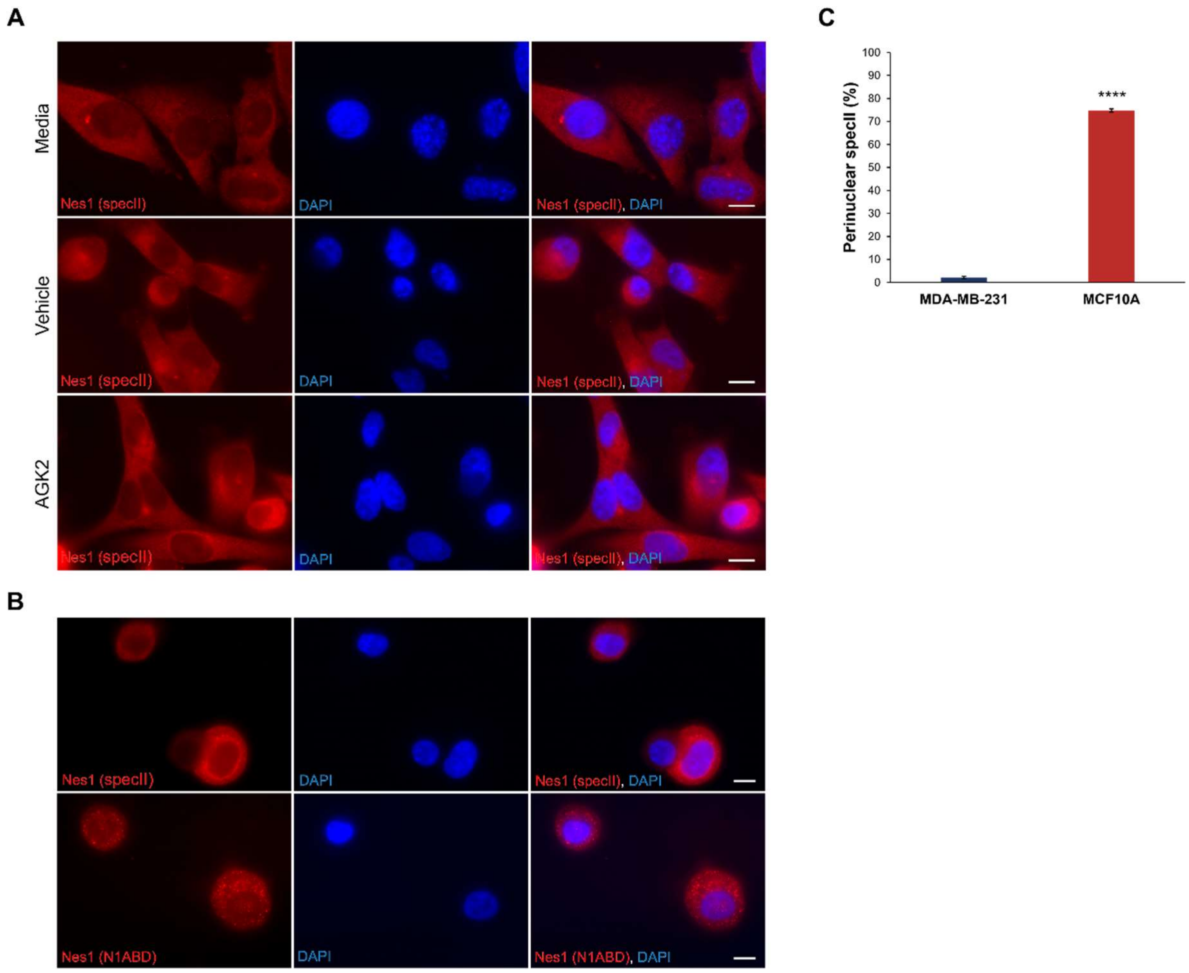

Supplement Figure 7: The localisation of Nesprin-1 differs between MDA-MB-231 and MCF10A cells. (**A**) Microscopy images of control and AGK2-treated MDA-MB-231 cells immunostained with the rabbit polyclonal specII antibody, which specifically targets the C-terminus of Nesprin-1 and encompasses spectrin repeats 73 and 74. Note the lack of prominent nuclear rim staining in all examined conditions. (**B**) Immunofluorescence microscopy images of MCF10A cells stained with the Nesprin-1 specII antibody (upper row) and Nesprin-1 N1ABD antibody (lower row). Nuclei are visualised using DAPI counterstain. Scale bar: 10 µm. (**C**) Quantification of the percentage (%) of MDA-MB-231 cells and MCF10A cells exhibiting prominent perinuclear specII staining; >300 cells analysed per cell type. The data are presented as the mean ± SEM, and statistical significance was determined using a Student's unpaired t-test. ****$p \leq 0.0001$ denotes statistical significance.

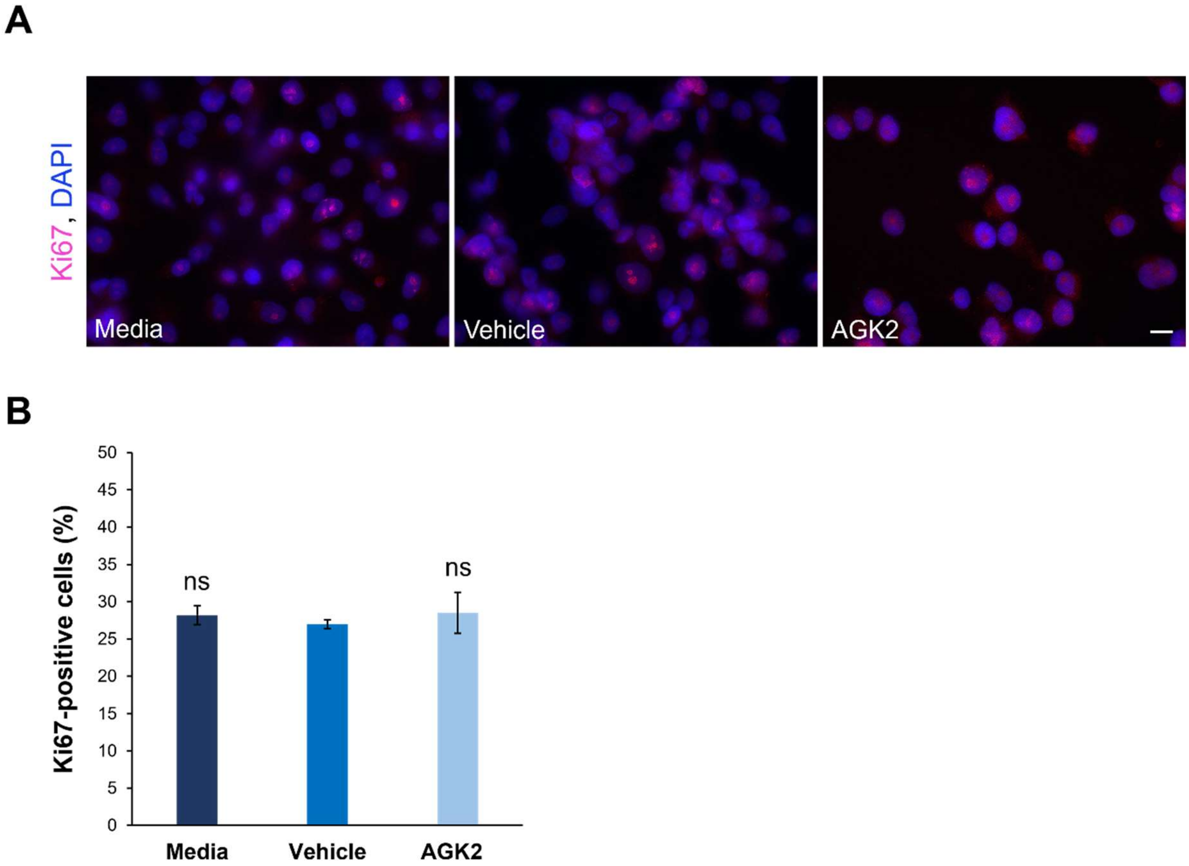

Supplement Figure 8: AGK2 treatment does not affect the proliferative capacity of MDA-MB-231 cells. (**A**) Representative fluorescence microscopy images of control and AGK2-treated MDA-MB-231 cells immunostained for Ki67, a well-established marker of proliferation in tumour cells. Scale bar: 10 μm. (**B**) Quantification of the percentage (%) of Ki67-positive MDA-MB-231 cells under control and AGK2-treated conditions; >700 cells analysed for each condition. The data is presented as the mean ± SEM. Statistical significance was evaluated using a one-way ANOVA to compare each treatment group to the vehicle control, with "ns" indicating non-significance.

Table S1. Primary antibodies for Western blotting

Antibody	Supplier	Product Code	Species	Dilution
SIRT2	Abcam	Ab67299	Rabbit	1:1,000
Acetylated histone 4	Abcam	Ab177790	Rabbit	1:1,000
Acetylated α-tubulin	In-house	-	Mouse	1:500
Vimentin [66]	In-house	-	Rabbit	1:400
GAPDH	Proteintech	60004-1-Ig	Mouse	1:10,000
β-tubulin	Proteintech	66240-1-Ig	Mouse	1:5,000
β-actin	Sigma-Aldrich	A5316	Mouse	1:5,000
Cytokeratin 8/18	Abcam	Ab17139	Mouse	1:5,000
Nesprin-1 (N1-ABD) [67]	In-house	-	Rabbit	1:1,000
Nesprin-2 (pAbK1) [68]	In-house	-	Rabbit	1:1,000
SUN1	Abcam	Ab124770	Rabbit	1:1,000
SUN2	Abcam	Ab124916	Rabbit	1:1,000
Lamin A/C (Jol2)	In-house	-	Mouse	1:20
Lamin B1	Abcam	Ab16048	Rabbit	1:1,000

Table S2. Secondary antibodies for Western blotting

Antibody	Supplier	Product Code	Species	Dilution
Anti-Rabbit HRP	Invitrogen	31460	Goat	1:5,000
Anti-Mouse HRP	Invitrogen	31430	Goat	1:10,000

Table S3. Primary antibodies for immunofluorescence staining

Antibody	Supplier	Product Code	Species	Dilution
Acetylated α-tubulin	In-house	-	Mouse	1:10
Vimentin [66]	In-house	-	Rabbit	1:400
Cytokeratin 8/18	Abcam	Ab17139	Mouse	1:500
Nesprin-1 (N1-ABD) [67]	In-house	-	Rabbit	1:100
Nesprin-2 (pAbK1) [68]	In-house	-	Rabbit	1:100
Nesprin-1 (N1 specII) [69]	In-house	-	Rabbit	1:100
Lamin A/C (Jol2)	In-house	-	Mouse	1:20
Ki67	LEICA	AK02	Mouse	1:100
GM130	BD Biosciences	610823	Mouse	1:200

Table S4. Secondary antibodies/ fluorescent stains for immunofluorescence staining

Antibody	Supplier	Product Code	Species	Dilution
Anti-Rabbit (Alexa Fluor™ 568)	Invitrogen	A11036	Goat	1:1,000
Anti-Mouse (Alexa Fluor™ 568)	Invitrogen	A11031	Goat	1:1,000
Anti-Mouse (Alexa Fluor™ 488)	Invitrogen	A21202	Donkey	1:1,000
**Fluorescent stains**				**Concentration**
TRITC-Phalloidin	Tocris	5783	-	20 ng/mL
DAPI	Sigma-Aldrich	62247	-	2 μg/mL
